# An overview on cardiac involvement in Inborn Errors of Metabolism: from clinical clues to nutritional management strategies

**DOI:** 10.3389/fcvm.2025.1648010

**Published:** 2025-12-04

**Authors:** Chiara Montanari, Veronica Maria Tagi, Martina Tosi, Eliana Stucchi, Eleonora Maria Pisano, Irene Raso, Giulia Fini, Laura Fiori, Savina Mannarino, Gianvincenzo Zuccotti, Elvira Verduci

**Affiliations:** 1 Department of Pediatrics, Vittore Buzzi Children’s Hospital, Milan, Italy; 2 Department of Biomedical and Clinical Science, University of Milan, Milan, Italy; 3 Department of Health Sciences, University of Milan, Milan, Italy; 4 Pediatric Cardiology Unit, "Vittore Buzzi" Children’s Hospital, Milan, Italy

**Keywords:** nutrition, metabolism, differential diagnosis, cardiac, dietary treatment

## Abstract

Inborn Errors of Metabolism (IEMs) account for a significant proportion of cardiomyopathies presenting with a wide spectrum of cardiac involvement, from isolated manifestations to multisystem syndromes. The manuscript explores the primary forms of cardiac complications linked to various IEMs, underlining the pathophysiological mechanisms and proposing a diagnostic framework guided by specific clinical features. Early diagnosis, especially in forms not captured by newborn screening, relies on careful clinical and metabolic evaluation. Thus, a multidisciplinary approach to address IEMs is essential, with the paediatric cardiologist contributing to both differential diagnosis and treatment. In addition, this review examines nutritional strategies for managing patients affected by IEMs with cardiac involvement, providing clinicians with research-backed guidance to support cardiological care, since specific nutritional strategies have shown promise in reversing or improving cardiac function in specific IEMs. Further research is needed to clarify the long-term safety of dietary interventions, particularly in pediatric populations, and to better define the role of nutrition in managing cardiomyopathies associated with IEMs, in order to develop evidence-based clinical protocols.

## 1 Introduction

**Figure 1:**
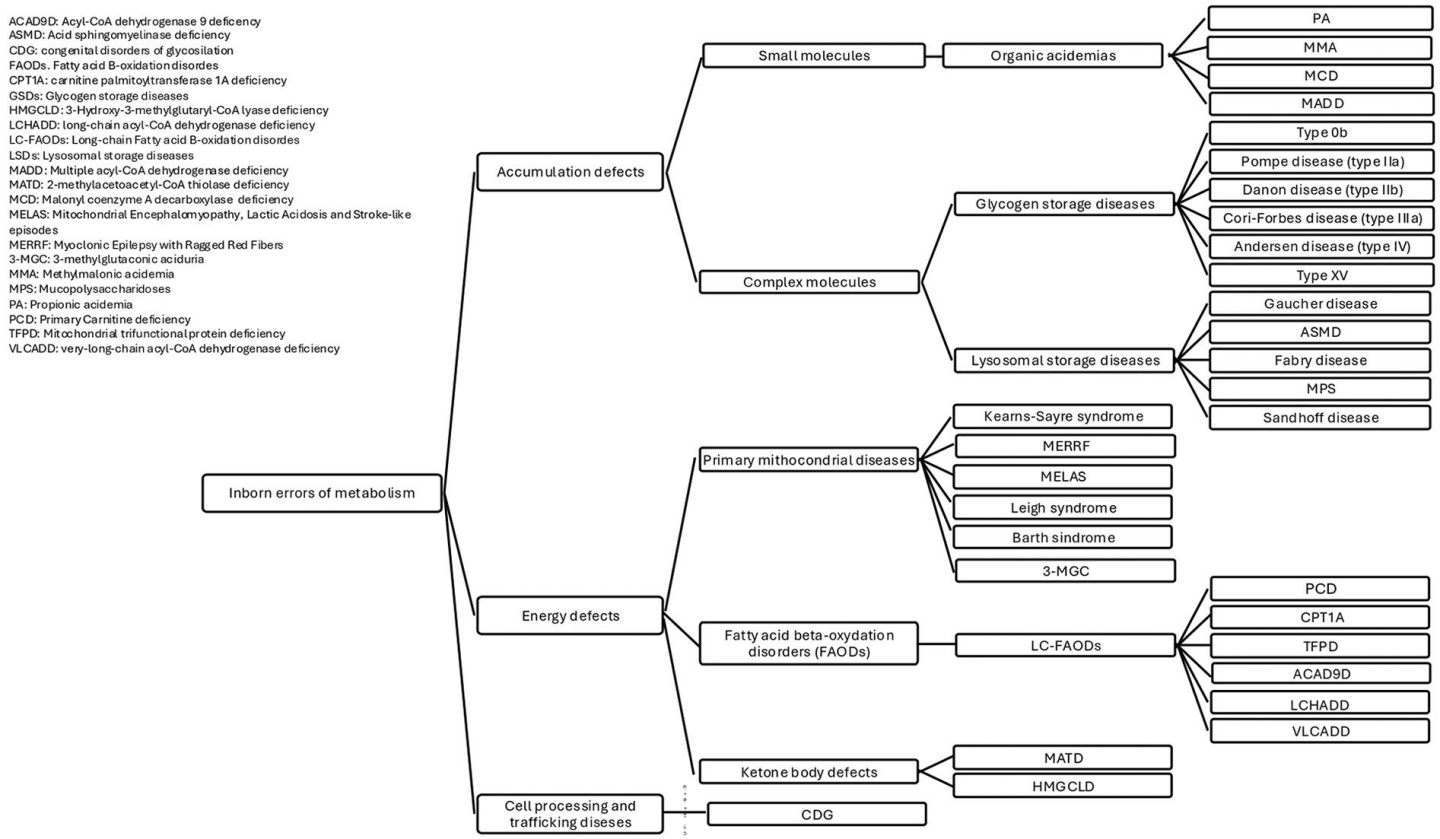
IEMs classification.

Approximately 5% of all Inborn Errors of Metabolism (IEMs) are associated with cardiomyopathy, a group of conditions characterized by abnormalities in the structure or function of the heart muscle (1). Cardiac disease may present as the first clinical manifestation or may develop during follow-up, varying in both pattern and severity, potentially progressing to heart failure (HF). IEMs affect cellular metabolic pathways including fatty acid oxidation (FAO), mitochondrial respiration and carbohydrate metabolism (2). The latest classification includes three main categories: “small molecules disorders”, “complex molecules disorders”, with subtypes based on accumulation or deficiency, and “energy defects”. Within the “accumulation type small molecule disorders”, some organic acidurias may present with cardiac involvement (3). Cardiac involvement is common in both muscular and hepatic glycogenosis and in several lysosomal storage disorders (LSDs), which are examples of “complex molecules disorders” with substrates accumulation. Within the “energy defects” category there are mitochondrial metabolic diseases that affect energy metabolism (3). Since the heart is an organ with high energy demands, it is frequently involved in fatty acid (FA) oxidation defects and disorders of the respiratory chain (3). Moreover, in the group of cell processing and trafficking defects of complex molecules, there are congenital disorders of glycosylation (CDG) (2, 3). They affect multiple systems, with phenotypical variability, including the cardiovascular system. Figure 1 provides a simplified classification of the diseases under discussion. Therefore, cardiac damage in IEMs may result from enzyme malfunction, leading to the accumulation of compounds harmful to the heart, energy deficiency, or impaired cardiac tissue development and function. Nutrition is a crucial aspect of care in patients affected by cardiological symptoms as the hemodynamic burden, progression of HF, and feeding intolerance often contribute to malnutrition (4), including those IEMs with cardiological involvement. Dietary treatment is essential for many IEMs. In recent years, various dietary regimens have been applied with the goal of both managing the disease and supporting cardiac function (5).

The aim of this narrative review is to describe the main types of cardiac involvement associated with IEMs, from a pathophysiological perspective, and to provide elements for a clinical differential diagnosis between IEMs. Secondly, we will focus on dietary treatments, offering recommendations on the management of cardiac involvement.

## 2 Methods

This narrative review was conducted with a systematic review approach following the PRISMA guidelines (Preferred Reporting Items for Systematic Reviews and Meta-Analysis) statement (6).

### 2.1 Data sources and search strategies

Firstly, a panel of Italian clinicians with expertise in IEMs, cardiology, and clinical dietetics was convened to identify IEMs with potential cardiac involvement. Subsequently, a PICO (Population, Intervention, Comparison, Outcome) strategy was developed. As summarized in Supplementary Table S1, two PECO/PICO questions were addressed “In patients with IEMs, what are the main types of cardiac involvement?” and “Which dietary treatments improve cardiac function in patients with IEMs and cardiac involvement compared to standard management?”. The literature search was conducted on two databases, namely PubMed/MEDLINE and Scopus. The complete search strategy terms are listed in Supplementary Table S2. The inclusion criteria consisted of only original studies written in English, with no restrictions on age or year of publication. Scientific articles, including systematic reviews, consensus statements, guidelines, observational studies, case reports, and case series, were included in the review.

### 2.2 Identification of relevant studies

Study selection was performed by four authors independently (double-blinded). After the removal of duplicate records, all articles were screened according to their titles and abstracts. Articles were excluded for the following reasons, namely background articles or irrelevant, wrong population, wrong publication type, and incorrect outcome. Afterwards, full text selection was performed, and in case of disagreement about the eligibility of any article, the opinion of a fifth author was sought.

### 2.3 Study selection and evaluation

Selected articles were fully analyzed to extract information about the main types of cardiac involvement in IEMs and the specific dietary treatments able to improve the cardiac function in patients with IEMs and cardiac involvement. We identified the following main outcomes: type and severity of cardiac manifestations, improvement of cardiac function or reduction in the progression of heart disease. Specific information is provided in Supplementary Table S1.

## 3 Results

**Figure 2:**
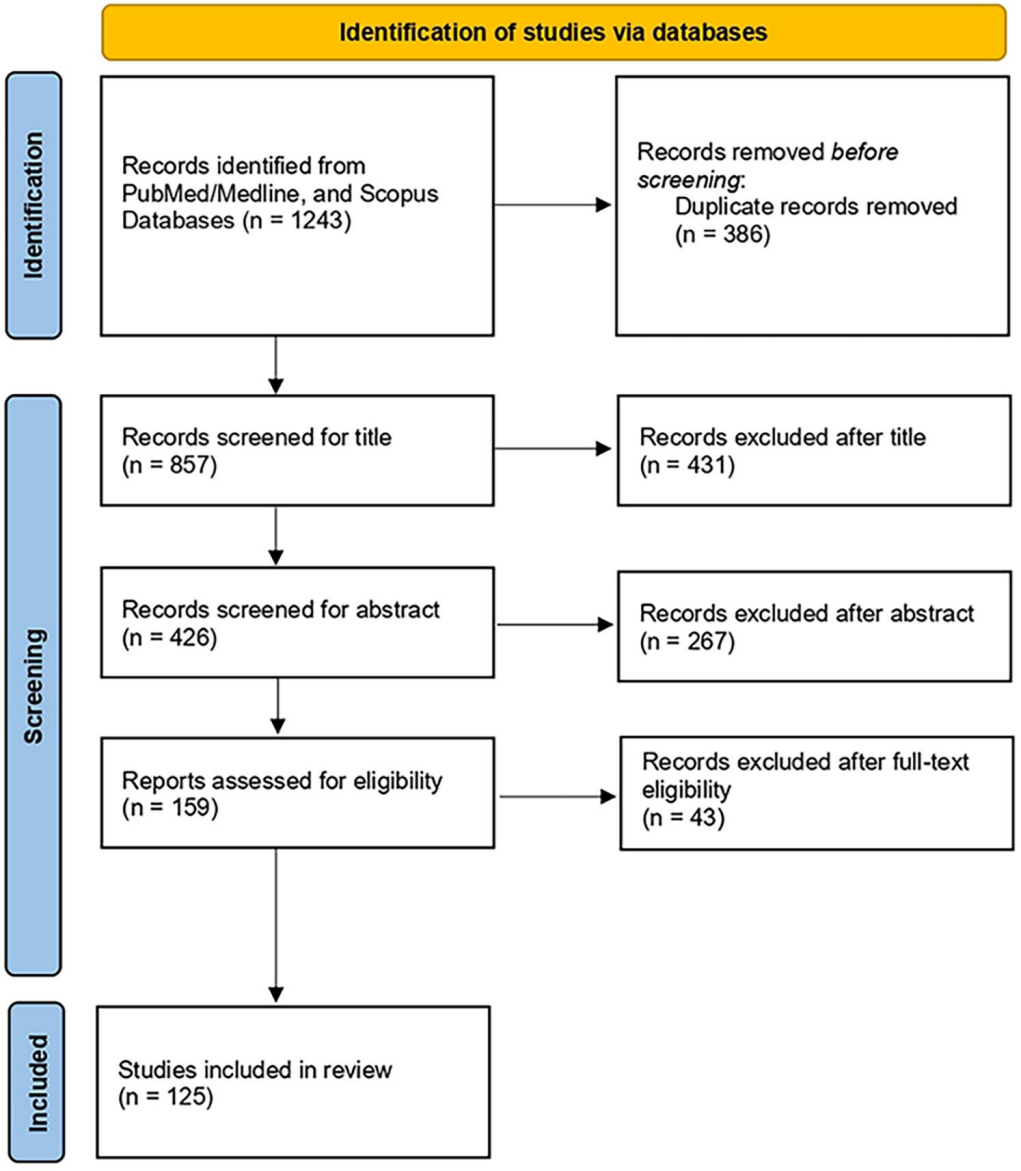
Flow diagram of the literature search process.

The initial systematic search retrieved a total of 1,243 articles published until 2025. After removal of duplicates, a total of 857 and 426 articles were screened for title and abstract, respectively. The remaining 159 articles were screened for full text. We finally included 125 articles. Figure 2 presents the flowchart of the review process.

### 3.1 Accumulation defects

#### 3.1.1 Small molecules

“Small molecule accumulation disorders” include amino acid catabolism disorders, urea cycle defects, organic acidurias, and galactosemia (3). These disorders have specific biomarkers, and after a symptom-free period, the accumulation of small molecules causes acute or progressive postnatal “intoxication,” worsened by food intake and catabolism. Dietary treatment aims to exclude the toxic compound and prevent catabolism. Rare conditions such as organic acidemias (OAs) lead to harmful metabolite accumulation in the heart and other tissues, leading to organ dysfunction. Clinical presentation may follow protein intake with acute neonatal onset, metabolic decompensation and neurological involvement, or follow a chronic course. Cardiomyopathies associated with OAs include dilated (DCM), hypertrophic (HCM), and left-ventricular non-compaction (LVNC) (3).

##### 3.1.1.1 Propionic and methylmalonic acidemia

*Background:* Propionic acidemia (PA) is a rare autosomal recessive (AR) disorder caused by deficiency of the mitochondrial enzyme propionyl-CoA carboxylase (PCC), necessary to convert propionyl-CoA to methylmalonyl-CoA (encoded by genes *PCCA* or *PCCB*). When PCC is deficient, propionyl-CoA and other harmful metabolites accumulate. Primary long-term effects include neurological issues, haematological disorders, hearing impairment, and heart problems like cardiomyopathy or acquired long QT syndrome (7, 8). Methylmalonic acidemia (MMA), closely associated with PA, is an AR disease caused by deficiency of the methylmalonyl-CoA mutase enzyme, which can be dysfunctional due to several different genetic defects (gene *MMUT* or one of the genes of intracellular cobalamin pathway (7). *MMUT* mutations have been historically classified as a mut0 subtype (no response to hydroxocobalamin supplementation) or a mut− subtype (responsive to hydroxocobalamin). Clinically, it presents with the same pattern of organ involvement (7).

*Cardiac involvement:* DCM is the form of chronic cardiomyopathy most linked to PA, cases of HCM, and LVNC have also been identified. However, cardiomyopathy can worsen quickly, potentially resulting in arrhythmias and HF. DCM can be the first sign in patients subsequently diagnosed with PA and cases where acute DCM was the sole presenting symptom of PA in both infants and adolescents were described (9, 10). Long QT syndrome (LQTS) is a chronic conduction disorder associated to PA (7). MMA is also known to cause cardiac dysfunction, though less commonly than in PA. Specifically, DCM and HCM are described, as well as acute complications as HF and ventricular fibrillation (1) leading to severe outcomes. Monitoring for cardiac issues in PA/MMA patients is mandatory (11).

*Dietary interventions:* In patients with PA/MMA, dietary management typically involves strict protein restriction to prevent the accumulation of toxic metabolites along with precursor-free amino acid and/or isoleucine/valine supplementation, as well as vitamin and mineral supplementation (7). Despite adequate total protein intake, patients with MMA and PA may show reduced levels of branched-chain amino acids (BCAAs), accompanied by elevated leucine/isoleucine (Leu/Ile) and leucine/valine (Leu/Val) ratios. Therefore, supplementation with Ile and Val is recommended to prevent high plasma Leu levels (12). Vitamin B12 treatment is recommended in responsive MMA patients. Long-term management aims to prevent metabolic decompensation and complications (13). A recent literature review examined potential treatment options for individuals who are either ineligible for liver transplantation or awaiting a transplant. Citric acid is a substrate that replenishes intermediates in the TCA cycle, enhancing its functioning. Since in rat hearts treated with high levels of propionate energy metabolism resulted impaired, citric acid has been proposed as a potential treatment, as it has been observed a notable rise in urinary levels of several TCA metabolites compared to baseline (8). However, there is no information available regarding the use of citric acid in PA patients with cardiomyopathy or long QT syndrome (14). Moreover, OXPHOS deficiencies and increased production of reactive oxygen species (ROS) have been observed in the hearts of PA patients with cardiomyopathy, indicating that oxidative stress might play a role in the development of cardiomyopathy (8, 15). Coenzyme Q10 (CoQ10), also known as ubiquinone, is essential for mitochondrial electron transport (8). Baruteau et al. suggest that low levels of coenzyme Q10 (CoQ10) in cardiac muscle could be a key factor in the development of heart complications in PA patients, resulting in secondary mitochondrial dysfunction (16). It also protects cell membranes from oxidative damage by inhibiting enzymes producing reactive oxygen species (ROS). Low CoQ10 levels in cardiopathic patients are linked to worse functional outcomes, including reduced left ventricular ejection fraction and higher N-terminal pro-B-type natriuretic peptide (NT-proBNP) levels (16). Severe HF correlates with lower CoQ10 levels in both plasma and myocardium (8). Correcting CoQ10 deficiency may help reverse cardiac symptoms in PA patients, offering insight into a potential therapeutic strategy for managing cardiomyopathy in this population (16). Oral supplementation of CoQ10 (ranging from 1.5 to 25 mg/kg/day) led to improvements in cardiomyopathy, but the authors did not confirm whether high dose CoQ10 supplementation restored myocardial CoQ10 levels (16). However, drawing definitive conclusions is challenging (8), and evidence supporting the use of CoQ10 remains limited (7, 13).

Under normal conditions, the heart primarily relies on fatty acids for energy but can switch to alternative substrates, such as ketone bodies (KBs), when needed (17). In HF, this metabolic flexibility is impaired, leading to disrupted energy metabolism and a shift in substrate utilization from fatty acids to glucose, which contributes to cardiac dysfunction in PA (18). KBs can serve as an alternative source of acetyl-CoA, supporting the TCA cycle when fatty acid or glucose metabolism is impaired. Baruteau et al. tested oral D,L-beta hydroxybutyrate (D,L-BHB) supplementation in a patient with PA and severe DCM, reporting improvements in heart function (with ejection fraction increasing from 10% at admission to 32% at discharge) (16). However, because other mitochondrial agents were administered simultaneously, the specific contribution of D,L-BHB remains uncertain (16).

Oral L-carnitine supplementation (100 mg/kg/day) is recommended to maintain normal plasma carnitine levels and support metabolic stability, although its effectiveness in restoring myocardial carnitine remains unclear (8). In addition, deficiencies in other micronutrients may contribute to mitochondrial dysfunction in PA. Proper functioning of the mitochondrial electron transport chain (mtETC) requires adequate levels of zinc, copper, selenium, and iron for adenosine triphosphate (ATP) production (13). The latest European Society of Cardiology (ESC) guidelines recommend supplementation for patients with identified nutritional deficiencies (19). Vitamin D and thiamine deficiencies may be associated with HF, but routine oral supplementation has not been shown to be effective. However, no data are currently available on the effects of micronutrient supplementation in PA patients with cardiomyopathy or LQTS (8). Current guidelines for treating patients with MMA and PA do not recommend specific therapies for cardiomyopathy or LQTS, other than standard cardiac treatments (7).

##### 3.1.1.2 Multiple acyl-CoA dehydrogenase deficiency

*Background:* Multiple acyl-CoA dehydrogenase deficiency (MADD), also known as glutaric aciduria type II, is an AR disorder affecting the electron transfer flavoproteins function (genes *ETFA*, *ETFB* and *ETFDH*) (20). They are involved in mitochondrial fatty acid beta-oxidation and in the delivery of electrons to the ubiquinone pool in the mitochondrial respiratory chain (21). The clinical manifestations can vary based on the age of onset. Neonatal form tends to be more severe, characterized by metabolic acidosis, non ketotic hypoglycemia, hyperammonemia, and cardiomyopathy (20).

*Cardiac involvement:* several studies have documented cases of chronic cardiomyopathy in MADD, such as DCM, left ventricular hypertrophy and acute complications as HF, especially in the severe neonatal-onset presentation. However, cardiac arrhythmias may occur also during metabolic decompensation in late-onset forms (20).

*Dietary interventions:* standard dietary treatment includes low-protein, low-fat diet, and the avoidance of prolonged fasting. Riboflavin can be used in responsive forms and CoQ10 and carnitine supplementation in secondary deficiency (20). Impaired energy metabolism often results in dysfunction across multiple organ systems, particularly under catabolic stress. Exogenous administration of KBs may help compensate for defective ketone production (22). Van Rijt et al. reported an improvement of cardiomyopathy in 70% of the presented cohort of 23 MADD patients, following treatment with D,L-3-hydroxybutyrate (22).

##### 3.1.1.3 Malonyl coenzyme A decarboxylase deficiency

*Background:* Malonyl coenzyme A (CoA) decarboxylase (MCD) deficiency is a rare AR organic acidemia, caused by mutations in gene *MLYCD*, in which malonyl-CoA is not decarboxylated to acetyl-CoA due to deficiency of the malonyl-CoA decarboxylase enzyme (23). It is characterized by high urinary excretion of malonic and methylmalonic acids and elevated malonyl carnitine. A dysregulation of fatty acid metabolism leads to similarities with FAO defects (24). The clinical picture may involve central nervous system (CNS) with developmental delay and seizures, metabolic acidosis, hypoglycemia, and cardiomyopathy (1).

*Cardiac involvement:* Cardiomyopathy is reported in 40% of patients and represents one of the leading causes of morbidity and mortality (25). Cardiac arrhythmias are often associated (24). In a study of 8 newly diagnosed patients, three cases of cardiac involvement were observed including cardiomyopathy, congestive HF, and mild left HCM. In addition HF resulting from decompensated HCM has been documented in a 2-month-old boy (1).

*Dietary interventions:* there is currently no consensus on the dietary management of MCD deficiency. The proposed rationale is based on a low-fat, high-carbohydrate diet, which has been associated with improvements in clinical condition, though biochemical responses and cardiac improvements remain inconsistent (24). Some evidence suggests that cardiomyopathy may improve with medium-chain triglycerides (MCTs), as these can be converted into KBs by the liver and used by the heart as an alternative energy. A long-chain triglycerides (LCTs)-restricted, MCTs-supplemented diet in a case of MCD deficiency with cardiomyopathy was successful in terms of improving cardiac function (24). Prada et al*.* described the first patient with MCD deficiency and LVNC. Although early dietary intervention did not prevent the onset of cardiomyopathy, restriction of LCTs combined with a high-MCTs/low-LCTs diet and carnitine supplementation led to improvements in cardiac function (26).

#### 3.1.2 Complex molecules

Some of the most prevalent IEMs involving the storage of complex molecules may present with marked cardiac manifestation as a noticeable cardiac thickening and enlargement, resembling HCM.

##### 3.1.2.1 Glycogen storage diseases

*Background and cardiac involvement:* Glycogen storage diseases (GSDs) are a group of IEMs that affect glycogen synthesis (glycogenesis) or breakdown (glycogenolysis), primarily in hepatic and/or muscle tissues. Depending on the underlying genetic defect and on the tissue-specific expression, deficient enzyme activity can result in fasting hypoglycaemia, hepatic dysfunction, hepatomegaly, myopathy, rhabdomyolysis, and cardiac manifestations, due to energy deficiency or the toxic effects of the accumulated glycogen (27). The main GSDs associated with cardiac involvement are described below:

- **GSD 0b** is an AR disorder caused by the loss-of-function mutations in glycogen synthase type 1 (gene *GYS1*), resulting in impaired glycogen biosynthesis in skeletal muscle and heart. Clinical manifestations include muscle weakness, myalgia and exercise intolerance, and HCM, which increases the risk of arrhythmia and cardiac arrest during exercise, even in the absence of prior exercise intolerance (27, 28, 152).
- **GSD IIa (Pompe disease, PD)** is an AR disease, caused by the deficiency of the enzyme acid alpha-glucosidase (gene *GAA*), which is responsible for the complete degradation of glycogen to glucose (2). Glycogen accumulation in lysosomes leads to cellular dysfunction, with impaired autophagy, affecting multiple tissues, particularly skeletal and cardiac muscles (29). Classic infantile-onset PD is the most severe form, and it is lethal without treatment. Within 6 months HCM, hypotonia, feeding difficulties, failure to thrive, and cardio-respiratory failure occur. Without enzymatic replacement treatment (ERT) survival rarely exceeds one year (29). Late-onset PD can present at any age, and it is characterized by muscular weakness and exercise intolerance. Cardiac involvement may occur as DCM or HCM, but it is milder, typically not appearing in the first year of life, and is generally absent in the adult form (2, 30, 31). Electrocardiographic abnormalities, such as short PR interval or increased amplitude of QRS complexes diffused in all leads, can also be observed (2, 30).
- **GSD IIb (Danon disease)** is a variant of PD with X-linked inheritance, characterized by severe cardiac manifestations and skeletal muscular weakness, caused by mutations of the gene *LAMP2* which encodes for a lysosome-associated membrane protein 2. A severe HCM, pre-excitation, and atrial fibrillation, varying degrees of atrioventricular block, and left bundle branch block can be observed. Cardiomyopathy tends to progress rapidly often leading to secondary dilatation and fibroelastosis, with potential need for heart transplantation (2, 32, 33). Danon disease can cause paediatric HF and appears to be underdiagnosed. Female carriers of *LAMP2* mutations are also susceptible to arrhythmias, HF and cardiomyopathy (33).
- **GSD IIIa (Cori-Forbes disease)** is an AR disorder caused by a deficiency of the glycogen-degrading enzyme amyloid-1,6-glucosidase (gene *AGL*). Abnormal glycogen accumulates between the myofilament, most commonly affecting the left or right ventricle and/or septal hypertrophy (34, 35). Typical clinical features include fasting ketotic hypoglycaemia, hyperlipidaemia, hepatomegaly, elevated transaminases, and, in some cases, mild periportal fibrosis or cirrhosis (27).
- **GSD IV (Andersen's disease)** is an AR disorder caused by loss-of-function mutations in the gene encoding a glycogen branching enzyme (*GBE*), resulting in abnormal glycogen chains storage in the central neuromuscular system and liver. The most severe clinical presentation occurs in the perinatal period and primarily involves neuromuscular function (2). After birth, affected children may develop severe cardiac involvement, including both HCM and DCM (2, 33) as well as hepatic dysfunction, hepatosplenomegaly, hypotonia, and failure to thrive. An adult-onset subtype presents as a neurodegenerative disorder (27).
- **GSD XV** is a rare AR disorder caused by mutations in the glycogenin type 1 (gene *GYG1*), an essential protein for the glycogen synthesis. Clinical manifestations include severe glycogen depletion in skeletal muscle and abnormal glycogen accumulation in the heart. Cardiac involvement typically presents as HCM, arrhythmia, systolic ventricular dysfunction, late-onset coronary artery disease, and HF (36, 37).

*Dietary interventions in GSD III:* regarding the impact of the diet on cardiac function, GSD IIIa is the most extensively studied. GSD IIIa has traditionally been managed with frequent meals throughout the day, rich in carbohydrates and proteins, aimed at reducing hypoglycemia. Guidelines recommend that 35%–55% of total energy be derived from carbohydrates, 20%–30% from proteins, and 20%–35% from lipids (38). Raw cornstarch may be used to maintain euglycemia, although even though lower doses are sufficient to maintain euglycemia compared to GSD I. A low intake of simple sugars in favour of complex carbohydrates and proteins is recommended. Alternatively, a nasogastric tube can be employed for nighttime enteral feeding, although this is less common than in GSD I (38). In adulthood, fasting tolerance improves, carbohydrates intake should be limited to prevent glycogen accumulation, while proteins intake may be increased (38). Additionally, a high-carbohydrate diet can induce hyperinsulinism, leading to activation of glycogen synthesis (34).

***High protein diet:*** a high protein diet provides an alternative source of glucose during fasting, enhances muscular protein synthesis and function, and promotes improvements in cardiomyopathy and creatine phosphokinase (CPK) levels (35). The benefits of this dietary approach have been described in some case reports by the literature. Dagli et al. reported the first patient with GSD IIIa and cardiomyopathy to benefit from a high-protein diet. This patient developed a severe left ventricular HCM at age 16. Following a dietary modification (increasing protein intake from 20%–25% to 30% of total energy and minimizing cornstarch to maintain normoglycemia), a marked improvement in the cardiomyopathy was observed (39). In adults, a low-calorie, high-protein diet has also been reported to produce significant improvements in electrocardiographic and echocardiographic hypertrophic indices. Specifically, a 900 kcal/day, high protein (37% of the total energy intake) was prescribed to a 32-year-old GSD IIIa patient, resulting in a 10 kg weight loss over 4 months. Subsequently, daily caloric intake was increased while maintaining a high-protein diet (43% of total energy), with sustained clinical stability (40).

***Ketogenic diet:*** cases in which the ketogenic diet (KD) has been used as a dietary approach in GSD III have been described in the literature, as KBs can serve as an alternative substrate for the heart and skeletal muscle. KD is characterized by a high fat and low carbohydrate content, with protein levels depending on the specific type of KD (41).

Marusic et al. described the case of a girl with GSD IIIa who was treated with a high—carbohydrate diet and developed left ventricular obstructive HCM, hepatomegaly and skeletal myopathy during her lifetime. Classical KD was introduced at the age of 11 years and maintained for over 4 years, resulting in normalization of left ventricular mass and improvement of hepatomegaly (42). Brambilla et al. described two siblings, 7- and 5-year-old, both affected with GSD IIIa, who developed left ventricular hypertrophy during the first year of life, while receiving adietary treatment characterized by frequent high-protein meals and uncooked cornstarch. After 12 months of high-fat (60%) and high-protein (25%), low-carbohydrate (15%) diet, congestive HF markers and symptoms improved, and thickness of interventricular septum and left ventricle posterior wall reduced (43). Moreover, an experimental treatment in a 2-month-old infant, consisting of a ketogenic (2:1), high protein (15%) diet, combined with a synthetic ketone body (D,L-3-OH butyrate), demonstrated an improvement in cardiomyopathy after 24 months of dietary treatment (44).

However, compliance with the KD is challenging due to the required fat to protein-carbohydrate ratio (4:1 or 3:1) at each meal and issue with palatability. A modified Atkins diet (MAD) has proven to be a valid alternative by Mayorandan at al., as more efficient and comfortable for the patient. It consists of limiting the daily carbohydrate intake to 10 g, free access to proteins and fats, and encouraging the last ones (34). Two boys with glycogenosis IIIa aged 9 and 11 years were treated, with an improved energetic state of heart and skeletal muscle, with a reduction of CK levels. Transient hypoglycaemia was observed. In one patient HCM resolved, in the other one left ventricular outflow tract obstruction significantly improved, brain natriuretic peptide levels normalized, and ST-elevation disappeared, even though left ventricle hypertrophy persisted (34). A 9-year-old boy with GSD IIIa with left ventricular hypertrophy diagnosed at the age of 4 years started a high-fat (50%), high protein (20%), low-carbohydrates (30%) diet. After 18 months, echocardiographic and biochemical parameters, including CK, showed improvement (35).

Rossi et al. reported on current experiences with lipid use in hepatic GSD III patients through an international, observational, retrospective multicentre cohort study of unpublished cases with different dietary lipid manipulations (45). Results show that cardiomyopathy and myopathy are the main indications to start a high-fat diet, with consequent CK levels decreasing, improved subjective strength in most of the patients, and cardiac hypertrophy improvement, the latter only in paediatric GSD IIIa patients (45). Lipid manipulation involved the use of MCTs in some GSD III patients. Some patients received both MCTs and a high fat diet, others MCTs supplementation (regular GSD III diet enriched in MCTs) or MCTs replacement (long-chain triglycerides substituted with MCTs) (45).

Lastly, Uçar et al. evaluated group of GSD IIIa patients with personalized high-protein (18.5%–28% of daily intake) and high-fat (70.5%–75.7% of daily intake) diet for 24 months (46). CK and lactate dehydrogenase levels significantly decreased, left ventricular mass and interventricular septum thickness improved and body muscle mass increased. Following an initial arrest in the height growth, subsequent improvement was observed by the end of the second year. Episodes of hypo- and hyper- glycemia decreased, and hepatosteatosis diminished by the end of the study, after an initial increase (46).

*Dietary interventions in PD:* in PD ERT enhances overall survival, cardiomyopathy and motor development in patients with infantile-onset PD, while helping stabilize the disease in late-onset form, improving motor function (29). To minimize glycogen buildup and muscle protein loss, a low-carbohydrate, high-protein diet has been suggested for these patients (29); if combined with exercise therapy, it helped slow the decline in muscle function in PD adult patients (47). Moreover, L-alanine supplementation has been suggested to lower muscle protein turnover and enhance muscular function (12, 48). A case study described the positive effects of L-alanine supplementation in an infant with PD, who presented at 12 months of age muscular hypotonia and developmental delay and at 30 months HCM with asymmetric septal hypertrophy and left-sided luminal obstruction (48). Oral L-alanine was supplemented for 5 years. Progression of skeletal myopathy was slow, and cardiomyopathy resolved almost completely (only mild septal hypertrophy at 5 years) (48). Another case of infantile onset PD girl ERT-treated since age 1 and supplemented with oral L-alanine was reported (49). After L-alanine supplement, fat mass gradually increased (low before) and resting energy expenditure gradually reduced from its initial high levels, suggesting an implementation of anabolic pathways (49). However, optimal nutritional intervention should be carefully assessed for each patient's nutritional status and modify their diet to fulfill their energy demands, as there is not enough data to suggest any one dietary plan beneficial for all patients with PD (29).

For the other types of GSDs, there is a dearth of official nutritional guidelines available, and no specific dietary treatment focused on cardiac involvement has been reported in the literature. The goals of individual care plans are to reduce glycogen buildup and hyperglycemia, and to prevent hypoglycemia and catabolism with subsequent hyperketosis in GSD IV patients. Traditional indicators of metabolic control, such as growth, liver size, serum aminotransferases, glucose homeostasis, lactate, and ketones, as well as liver function are typically balanced with symptoms. Cardiac monitoring is also advised (ECG and echocardiogram) (50).

##### 3.1.2.2 Lysosomal storage diseases

Lysosomes function as the cell's recycling centers, breaking down substances that are brought into the cell and also clearing away cellular waste (5). LSDs result from a lack of enzymes needed for degradation, causing the abnormal accumulation of incompletely catabolized substrates within organelles, with a progressive impairment of cell function (i.e., connective tissue, solid organs, nervous system) (2). We will focus on cardiac involvement in Gaucher disease (GD), Acid Sphingomyelinase deficiency (ASMD), Fabry disease, GM2 gangliosidosis (Sandhoff disease) and mucopolysaccharidoses (MPS) diseases. In LSDs, ERT is essential, but specific dietary recommendations can also be applied, even though, to date, there are few available data on their impact on cardiac function.

##### 3.1.2.3 Gaucher disease

*Background:* Gaucher disease (GD) is an AR disorder caused by mutations in the gene *GBA1*, which encodes the enzyme acid beta-glucosidase, leading to the accumulation of glucosylceramide mainly in liver, spleen and bone marrow (5). GD I involves multiple systems without CNS involvement, whereas GD II and III primarily affect the CNS (2). A cardiovascular form (GD IIIc), is mainly caused by p.D409H mutation of the gene *GBA1* (51).

*Cardiac involvement:* Pulmonary hypertension, cor pulmonale, and pericarditis due to intrapericardial hemorrhage have been reported in GD (51). GD IIIc differs from other GD types as it is mainly characterized by valvular heart disease. However, since fewer than 50 cases of GD IIIc have been reported so far, the full range of its clinical features is still not well understood (52). In addition to aortic and mitral valve regurgitation or stenosis, HCM or DCM, myocardial fibrosis, tachycardia, atrial fibrillation, and bradycardia have also been reported (2) likely linked to myocardial infiltration by typical Gaucher cells (53).

*Dietary interventions:* ERT or substrate reduction therapy (SRT) is advised for all symptomatic patients, as these therapies greatly improve many symptoms of GD I (29). Patients with GD I often experience an increased resting energy expenditure and are at risk of malnutrition. Resting energy expenditure normalizes with ERT, which may lead to weight gain (54). Thus, careful monitoring and modification of dietary plans to maintain a balanced nutritional status is essential (54). Nascimbeni et al. described glucose/lipid metabolism in adult GD I adult patients (55). Even though adults' GD I present peripheral insulin resistance, reduced HDL-cholesterol, increased triglyceride and apolipoprotein E plasma levels, no increased incidence of type 2 diabetes and premature atherosclerosis was reported. However, cardio-cerebrovascular events can be the cause of death in treated GD I patients (29, 55). Lipid profiles normalize in GD I patients with ERT (29). Other nutritional recommendations are limiting consumption of disaccharides, in case of gastrointestinal issues like diarrhea, abdominal pain or discomfort, due to SRT miglustat treatment, and avoid fruits and vegetables that affect cytochrome P450, CYP2D6 and CYP3A metabolism altering eliglustat plasma levels, an available SRT (5, 29). No dietetic approach related to cardiac form (GD IIIc) has been reported in the literature.

##### 3.1.2.4 Acid sphingomyelinase deficiency

*Background:* Acid sphingomyelinase deficiency (ASMD) is a neurodegenerative metabolic lipidosis that leads to the accumulation of sphingolipids in the body's cells. It is an AR disease caused by different mutations in the gene *SMPD1* (5). ASMD is classified into infantile neurovisceral (ASMD A), chronic neurovisceral (ASMD A/B) and chronic visceral (ASMD B) (56). ASMD non-neurological symptoms include lung disease, hepatosplenomegaly, liver dysfunction, thrombocytopenia and coagulation defects, dyslipidemia, osteopenia and growth delay (30).

*Cardiac involvement:* although cardiac involvement is rare, endocardial fibrosis is described (53). Cardiac dysfunction is observed in patients with advanced ASMD, either independently or secondary to pulmonary disease (5, 57). In a cross-sectional study of 59 patients, abnormalities in electrocardiograms and echocardiograms were seen in 28% and 50% of cases, respectively (57). Valve abnormalities are also described (5, 57). Plasma lipid imbalance is also prevalent in ASMD, with an atherogenic lipid profile. Additionally, hypertrophy of smooth muscle cells in the medial and intimal layers of distal coronary arteries has been noted, contributing to the apparent acceleration of atherosclerosis in these patients (57).

*Dietary interventions:* although specific nutritional guidelines for ASMD patients are lacking, assessing their nutritional status is crucial. Tailored dietary plans should be designed to meet the patient's needs, considering their resting energy expenditure, especially in late adolescence and adulthood to ensure proper nutrition and help manage hyperlipidaemia. Additionally, it would be valuable to assess the actual impact of a low-fat diet in these patients (29). No dietetic approach focused on cardiac involvement has been reported in the literature.

##### 3.1.2.5 Fabry disease

*Background:* Fabry disease (FD) is an X-linked recessive disorder caused by mutations in the gene *GLA*, which lead to a deficiency in *α*-galactosidase A. This results in the accumulation of globotriaosylceramide (GL-3, Gb3) and globotriaosylsphingosine (lyso-GL-3) within lysosomes, affecting mainly cardiac cells, blood vessels, kidneys, nerves, causing their dysfunction (5). The classical form of Fabry disease primarily affects males, with symptoms such as chronic neuropathic pain, angiokeratomas, chronic kidney disease (CKD), cardiovascular issues and gastrointestinal problems. The late-onset form of Fabry disease is more common, with typical cardiac symptoms and proteinuria (29).

*Cardiac involvement:* chronic cardiac manifestations include HCM, especially involving the interventricular septum and the posterior left ventricle, increased endomyocardial trabeculation, mitral insufficiency, aortic and mitral valve thickening (2, 33). Lipid accumulation in conduction tissues leads to bradyarrhythmias or atrial flutter. ECG often shows a short PR interval, and signs of biventricular hypertrophy. Lipid deposition in the coronary arteries can lead to ischemia and myocardial infarction (58). Skeletal muscle involvement is generally milder and occurs later. Cardiac complications are the leading cause of early mortality in both affected men and women (33). While cardiac involvement has been observed in patients with various mutations, the mutation p.N215S (c.644A > G) in exon 5 of the gene *GLA* has been identified as the cause of primarily cardiac symptoms, particularly late-onset left ventricular hypertrophy, atrial tachycardia, and conduction abnormalities (2).

*Dietary interventions:* although there is insufficient strong evidence to recommend a specific dietary plan for patients with FD, a low-FODMAP (fermentable oligosaccharides, disaccharides, monosaccharides and polyols) diet relieves gastrointestinal symptoms, since recent studies have shown that short-chain fermentable carbohydrates increase water content in the small intestine and gas production in the colon (5, 29). ERT is a recognized treatment for FD, either alone or in combination with chaperone therapy (migalastat), and it helps prevent or delay the progression of certain complications like the increase in left ventricular mass (2). In patients with CKD, a low-protein diet supplemented with ketoanalogues may help slow the progression of CKD (5, 29). No dietetic treatment focused on cardiac involvement has been reported in the literature.

##### 3.1.2.6 Mucopolysaccharidoses

*Background:* mucopolysaccharidoses (MPS) are a group of disorders caused by the deficiency of enzymes involved in the breakdown of glycosaminoglycans. Due to the rare and variable nature of MPS, diagnosis can be challenging. Symptoms can include intellectual disability, facial abnormalities and a multisystemic organ involvement, leading to hepatosplenomegaly, skeletal abnormalities, corneal clouding, lung disease, hearing problems and cardiac disease (29). The heart and blood vessels are frequently involved. Although heart-related issues can be seen across all types of MPS, they are more prevalent in MPS I, II, and VI (59). In the most severe form of MPS I, known as Hurler's disease, patients die within the first ten years of life (60). In contrast, the clinical presentation of the attenuated forms of MPS I (Hurler-Scheie and Scheie syndromes) is much more varied, with significant phenotypic differences (29). MPS III, or Sanfilippo syndrome, is a primarily neurodegenerative condition (29).

*Cardiac involvement:* heart complications tend to emerge at a younger age in individuals with faster-progressing variants, whereas they may appear later in slowly advancing forms (59). MPS I can present both HCM and DCM, MPS VI primarily presents with DCM alone, whereas MPS II, III, IV and VII can present with HCM (33). Thickening of the cardiac valves and large vessels has been observed in MPS I patients, with the left-sided valves typically more severely affected (53). Cardiac valve involvement is more common in syndromes where the breakdown of dermatan sulphate is disrupted (59). Coronary artery narrowing or occlusion have been observed in individuals with all types of MPS, though they are most seen in MPS I and MPS II. Radiological examination typically shows generalized cardiomegaly and calcification of the mitral valve ring. There are no specific electrocardiographic abnormalities (53). Ventricular septal defect closure has been also described in a 15 year old patient (59).

*Dietary interventions:* although research on nutrition in MPS patients is limited, malnutrition remains a concern, as well as the effects of diet and lifestyle on cardiovascular and bone health. The development of specific ERT has significantly improved the quality of life and life expectancy for patients with certain types of MPS. Nutritional monitoring and guidance are recommended for MPS patients, as recent studies found that those with MPS I, II, or VI often have inadequate intake of energy and micronutrients, potentially affecting disease progression (29). Supplementation with vitamins B1, B2, B3, vitamin C and iron can be useful (5). Vitamin D should be supplemented in case of deficiency and bone disease, for the risk of fracture (29). Studies in MPS animal models have indicated that a fat-rich diet could be beneficial (61, 62). While the isoflavone genistein has shown potential in inhibiting glycosaminoglycan synthesis and accumulation, its supplementation in MPS I mice resulted in unexpected adverse effects. Clinical trials are still ongoing, and insufficient data are available to endorse this treatment (63). CoQ10 in lysosomal membranes plays a crucial role in electron exchange, helping with proton translocation and the acidification of the lysosomal environment. Deficiencies in CoQ10 and pyridoxal phosphate (PLP) have been found in most patients with MPS III. *In vitro* CoQ10 supplementation reduces glycosaminoglycan accumulation in Sanfilippo A and B cell lines (64). Supplementation with CoQ10 and PLP could potentially be beneficial as an adjunctive treatment, though more research in humans is needed (65). No specific dietetic treatment focused on cardiac involvement has been reported in the literature.

##### 3.1.2.7 Sandhoff disease

*Background:* Sandhoff disease is an AR LSD caused by mutations in the gene *HEXB*, which encodes for the beta subunit of the enzyme hexosaminidase. Ganglioside GM2 accumulates abnormally in various tissues, leading to progressive central nervous system degeneration and early blindness, in addition to hepatosplenomegaly, macrocephaly, and cherry-red spots on the macula (66).

*Cardiac involvement:* although less common, there have been reports of cardiac issues in nine infantile patients with Sandhoff disease. These patients exhibited mitral valve prolapse and regurgitation, atrial and ventricular septal defects, DCM and HF. Some also showed heart murmurs and mild arrhythmias from atrioventricular block (AVB), along with asymmetric hypertrophy of the interventricular septum (66).

*Dietary interventions:* in addition to Substrate Reduction Therapy (SRT) with miglustat, which may help slow the neurological progression, a KD demonstrated an improvement in motor behavior and longevity in Sandhoff disease mouse models. In combination with miglustat, KD had a potential additive effect, resulting in increased delivery of miglustat to the central nervous system (67). Villamizar-Schiller and al. described a case of a 6-year-old affected by Sandhoff disease with DCM (66). Ventricular function was borderline with an ejection fraction of 50%. The patient's heart function improved after starting treatment with miglustat and a KD (dietary fat percentage was increased from 30% to 80% of his total caloric intake). Echocardiogram, performed 12 months after starting KD and miglustat, revealed a reduction in ventricular enlargement with an improvement of ejection fraction to 72%. In addition, seizures were better controlled, and the liver was no longer palpable (66).

### 3.2 Energy defects

#### 3.2.1 Primary mitochondrial diseases

*Background and cardiac involvement:* primary mitochondrial diseases (PMDs) are a heterogeneous group of multisystem disorders due to anomalies in the mitochondrial respiratory chain or cellular oxidative phosphorylation (5), caused by mutations in nuclear or mitochondrial DNA. It can be diagnosed at any age due to its wide range of clinical presentations, though more severe forms typically appear in infancy (33). The phenotypic variability depends on the level of heteroplasmy (mtDNA) (5). Here, we discussed selected PMDs. Generally, structural and functional defects as HCM, DCM, and left ventricular noncompaction (LVNC) are described, as well as arrhythmias which can worsen during an acute metabolic decompensation (5).

- **Kearns–Sayre syndrome (KSS):** it is mostly caused by deletions in mitochondrial DNA and it is characterized by ptosis, chronic progressive external ophthalmoplegia, abnormal retinal pigmentation, cardiac conduction defects and DCM (33). Bundle–branch blocks, fascicular blocks, and nonspecific intraventricular conduction disturbances are described in KSS and can lead to recurrent syncope or sudden death (33).
- **Myoclonic epilepsy with ragged red muscle fibers (MERRF) syndrome**: it can be caused by mutations in different mitochondrial genes, among which mutations in the gene *MTTK* is the most frequently reported. *MTTK* encodes a tRNA molecule that binds L-lysine, called tRNALys, which is responsible for the incorporation of lysine during protein synthesis (33). MERRF is characterized by myoclonus, epilepsy, ataxia and weakness, hearing loss, short stature, optic atrophy, and cardiomyopathy with WPW (Wolff–Parkinson–White) syndrome. Thus, patients with MERRF should be monitored for the development of HCM and DCM (33).
- **Mitochondrial encephalomyopathy, lactic acidosis, and stroke-like episodes (MELAS)**: it is a syndrome most commonly caused by mutations in the mitochondrial gene *MTTL1,* which encodes tRNALeu (68). Although cases of left ventricular noncompaction (LVNC) have been reported in MELAS, cardiomyopathies are rarely described in pediatric patients with this syndrome, as cardiac involvement typically emerges later in life. Thus, MELAS patients require regular monitoring for potential onset of HCM and DCM. Common features in MELAS patients include short stature, epileptic seizures, unilateral weakness, visual field defects, and vision loss (33).
- **Leigh syndrome (LS):** it is the most common infantile mitochondriopathy. It is a progressive neurodegenerative disorder with a genetic heterogeneity regarding the other organ involvement (33). LS is characterized by marked genetic heterogeneity, with around 60 genes implicated to date, the majority located in nuclear DNA, while about 25% are found in mitochondrial DNA. These mutations impact all five complexes of the mitochondrial respiratory chain (33). Left ventricular hypertrophy is the dominant echocardiographic sign in LS (10). However, cardiomyopathy can be hypertrophic, as well as dilated, and conduction defects, such as WPW syndrome, can be found (33).
- **Barth syndrome (BTHS):** it is a rare X-linked genetic disorder (gene *TAZ*) characterized by muscular weakness, short stature, cardiomyopathy, hypocholesterolemia, cognitive impairment, intermittent neutropenia and biochemically by 3-methylglutaconic aciduria. Gene *TAZ* encodes tafazzin, a protein involved in the remodeling of cardiolipin, an essential lipid of the inner mitochondrial membrane (33). BTHS is a secondary 3-methylglutaconic (3-MGC) aciduria, which differs from primary 3-MGC aciduria by the accumulation of this marker due to mitochondrial dysfunction, rather than a genetic defect in the enzyme responsible for its metabolism (69). In a recent UK study cardiac dysfunction was the main symptom of patients with BTHS, half of them diagnosed during the first month of life, with a high mortality during infancy (69). It is associated with DCM and with isolated LVNC. A myocardial remodeling process over time can be observed through echocardiography, from both dilated and hypertrophic form with hyperdynamic function to dysfunction (33). It is important to underline that patients may go through a “honeymoon period” after the age of 3, during which heart function improves and morbidity declines. But by the age of ten they experience deteriorating cardiomyopathy, chronic fatigue, diminished exercise ability, and potentially lethal arrhythmias (69). However, there is a high phenotypic variability and young males without any additional BTHS symptoms who only showed cardiomyopathy should be investigated for BTHS (5).

*Dietary interventions:* various non-pharmacological treatments aimed at enhancing mitochondrial function and improving prognosis have been explored, with a particular focus on the KD. In patients affected by Leigh encephalopathy and MELAS syndrome, a clinical improvement, including cardiac health, has been proved (5). Specifically, KD is a treatment option in patients with PMDs with encephalopathy and refractory seizures, but it can be a therapeutic option also in case of cardiomyopathy, even though long term complications of KD can include cardiovascular disease due to dyslipidemia (70).

Kucharska et al. described a male infant diagnosed with LS presenting with HF due to a severe progressive HCM unresponsive to cardiological pharmacotherapy, mitochondrial cocktail therapy and mechanical ventilation. KD has led to a significant clinical and echocardiographic improvement with no adverse effects reported (70).

Similarly, Deberles et al. described a 3-year-old girl with mitochondrial disease (mutation m.5559A > G in the mitochondrial gene tRNATrp) with septal ventricular hypertrophy (71). KD was introduced in association with antioxidant supplementation. Not only the neurologic status improved but also cardiological parameters, with normalisation of interventricular septum thickness at 6 years of age (71).

A systematic review of the literature aimed to evaluate the safety and efficacy of KD for PMDs. KD was effective in improving myopathy in 3 of 10 individuals, and in reversing cardiomyopathy and movement disorders in 4 of 20 cases, as well as in controlling seizures (72). While KD may be considered for patients with MD and therapy-refractory epilepsy, there is currently insufficient information on the safety and effectiveness of KD for MD to make broad recommendations. KD is contraindicated in myopathies associated with mitochondrial DNA deletions (72).

#### 3.2.2 Fatty acid beta-oxidation disorders

The most common underlying mechanism of cardiac involvement in FAO disorders and carnitine deficiency is the disruption of energy generation. Cardiac symptoms usually dominate the clinical phenotype, have an abrupt onset, and present early in the course of the disease. Additionally, their identification frequently results in the diagnosis of the underlying congenital disease (10). Long-chain fatty acids bind to carnitine in the cytosol and are transported into the mitochondria, where they undergo beta-oxidation, breaking down the fatty acids into smaller units that enter the tricarboxylic acid cycle for energy production. This process is crucial during fasting, supplying energy to skeletal and cardiac muscle, the liver, and other tissues (73). During fasting KBs are typically the primary energy source for tissues like the heart (74). Long-chain fatty-acid oxidation disorders (LC-FAODs) are AR IEMs resulting in interference with long-chain fatty-acids oxidation or their entry into the mitochondria (73). Clinical issues caused by both the toxicity of these fatty acid esters and energy production problems. The toxic buildup affects cardiac muscle, causing damage to the cardiac myocytes (74). The severity of clinical presentation may range from critical illness in infants, including hypoketotic hypoglycaemia, liver dysfunction, cardiomyopathy, skeletal myopathy, rhabdomyolysis, and sudden death, to exercise intolerance in adults. Newborn screening permits an early diagnosis preventing decompensation during acute illness or fasting, also in mild forms (73).

#### 3.2.3 Primary carnitine deficiency (PCD)

*Background:* PCD, an AR disorder caused by mutations in the gene *SLC22A5*, prevents carnitine transportation into the cells, which is essential for shuttling long-chain fatty acids into the mitochondria (75). Thanks to newborn screening, mild forms can be early diagnosed and treated and, since carnitine is transported across the placenta during pregnancy, low carnitine levels on newborn screening may actually reflect a previously unrecognized maternal disease (75). A screening program conducted in the Faroe Islands in asymptomatic or paucisymptomatic patients revealed the highest prevalence reported in the world (1:300) in adult patients (15–80 years) (76).

*Cardiac involvement:* in PCD, echocardiography showed LVNC, normal dimensions apart from left ventricular hypertrophy with normal systolic function in one young male. Electrocardiograms showed mild abnormalities. However, asymptomatic patients who are not treated during periods of physiological stress may be at risk for decompensation (10). DCM and HCM can be a clinical manifestation in children with PCD, with an age of onset of 2–4 years of age. Although DCM appears to be more common than HCM, some patients who present with DCM may also have modest ventricular hypertrophy (10). Arrhythmias such as cases of QTc prolongation in children with PCD have been described (10, 76), since energy production is essential also for heart conduction.

*Dietary interventions:* in PCD, symptoms such as fatigue and palpitations were reported in 43% and reduced to 12% after L-carnitine supplementation. Unfortunately, control echocardiographic tests in adult patients after a prolonged L-carnitine supplementation are not available (76). However, although serious symptoms appear to be rare in PCD, L-carnitine supplementation is recommended to avoid an increased risk of cardiac arrhythmias (76).

#### 3.2.4 Long-chain fatty-acid transport defects

*Background:* Long-chain fatty acids must first bind to carnitine to be transported inside mithocondria. This process is catalyzed by the enzyme Carnitine Palmitoyltransferase 1 (CPT-1, encoded by the gene *CPT1A*), located on the outer mitochondrial membrane, which forms acylcarnitines. These are then transported across the inner mitochondrial membrane by Carnitine Acylcarnitine Translocase (CACT, encoded by the gene *SLC25A20*). Inside the mitochondria, Carnitine Palmitoyltransferase 2 (CPT-2, encoded by the gene *CPT2*), located on the inner membrane, detaches carnitine from the fatty acids, regenerating acyl-CoA, which enters *β*-oxidation (77). CPT-1 deficiency is a AR disorder characterized by an increased risk of hypoglycemia, liver failure, hepatic encephalopathy and hyperammonaemia, particularly during periods of fasting or illness (73). CACT deficiency (AR) is similar to CPT-1 deficiency with also muscle stiffness and heart disease (73). CPT-2 deficiency (AR) is categorized into 3 forms: a myopathic form, a severe infantile form (recurrent hypoglycemia, liver dysfunction, rhabdomyolysis and cardiomyopathy) and a lethal neonatal form (73).

Cardiac involvement: cardiac involvement is very rare in CPT1A deficiency. In contrast, cardiomyopathy and heart rhythm disorders have been described in CACT deficiency and in CPT2 deficiency (10, 73). A multi-center international retrospective review described 23 patients affected by CACT deficiency. Of 16 classical cases, 15 presented cardiac involvement with cardiac arrhythmias (9/15), cardiac arrest (7/15), and cardiac hypertrophy (9/15) (78).

*Dietary interventions:* the general management of these disorders involves avoiding prolonged fasting and reducing lipid intake, particularly long-chain fatty acids (79). No specific dietetic treatment focused on cardiac involvement has been reported in the literature.

#### 3.2.5 Long-chain fatty-acid oxidation defects

*Background:* very-long-chain acyl-CoA dehydrogenase deficiency (VLCADD) is caused by two pathogenic mutations in the gene *ACADVL,* encoding for the enzyme very-long-chain acyl-CoA dehydrogenase. Three different phenotypes have been described for VLCAD deficiency: the severe early-onset form with cardiac and multiorgan failure, the hepatic childhood form characterized by hepatomegaly and hypoketotic hypoglycemia, and the late-onset muscular form presenting with episodes of rhabdomyolysis (80).

Long-chain 3-hydroxyacyl-CoA dehydrogenase deficiency (LCHADD) and trifunctional protein (TFP) deficiency are two fatty acid beta-oxidation disorders, caused by biallelic pathogenic variants of the genes *HADHA* and/or *HADHB*, molecularly differentiated but clinically indistinguishable. They present with hypoglycemia, hepatic dysfunction, cardiomyopathy and sudden death (73).

*Cardiac involvement:* VLCADD affects both skeletal and cardiac muscle. Cardiomyopathy is more commonly linked to defects involving the oxidation of long or very long chain fatty acids, where infantile HCM is the most common clinical phenotype (10) with an increased risk for sudden death. With the expansion of newborn screening, forms with a milder phenotype have been more commonly diagnosed, with easy fatigability or no clinical symptoms reported until adolescence or adulthood (73). Prolonged QTc and ventricular tachycardia have been found in infants with VLCADD (10). An infant with VLCADD deficiency but without diagnosis yet, presented severe cardiac manifestations at the age of 5 months, including massive pericardial effusion, in addition to HCM (81). Kwiatkowska et al. described 17 children diagnosed affected by LCHADD, confirmed genetically, at the mean age of 6 months, under a regular follow-up. Cardiac abnormalities were detected in 15 of 17 patients (74). Cardiomyopathy in 11 children, and, in 2 cases an unusual evolution from DCM into HCM was described. The other cardiac anomalies observed include an intermittent ventricular preexcitation on 24 h Holter-electrocardiography, prolongation of QTc and nonspecific repolarization abnormalities. Additionally, coarctation of the aorta was found in one patient (74). Other studies reported a variable percentage of cases of cardiomyopathies: 42% in a cohort of 50 patients diagnosed with LCHADD (82), 26.2% in a cohort of 107 patients (83) and 61.5% in a cohort of 16 patients (84). Moreover, a cardiac tamponade that had to be drained was found in a patient at diagnosis of LCHADD (84).

*Dietary interventions:* current dietary management in LC-FAODs is based on avoiding prolonged fasting, restricting long-chain fatty acid (LCFA) intake, supplementing with MCTs, and moderately increasing carbohydrate intake (73). Dietary fat should be limited to 20%–30% of the total energy intake (73). Moreover, essential fatty acids should be monitored and supplemented as needed. Patients with severe LC-FAODs may require a 2:1 ratio of MCTs to LCFA. The most severe disorders present significant cardiac or hepatic involvement shortly after birth, thus the severity of the disorder often is correlated with the timing of first decompensation (73). On the contrary, fats can be slightly more freely consumed by asymptomatic patients, accounting for 30%–40% of total energy and only 10%–15% of MCTs (85).

The standard treatment for VLCADD patients has been designed to restrict LCTs and supplement with MCTs to bypass impaired long-chain FAO and enhance KBs production in the liver (86). The real effectiveness of adequate dietary treatment on cardiac function was described in the clinical case reported by Kim et al. (81). An infant was diagnosed with VLCADD at the age of 5 months when presented with a massive cardiomegaly, HCM and pericardial effusion. After diagnosis, a high concentration of intravenous dextrose, 50 mg riboflavin per day, and an MCTs formula was administered. Echocardiography revealed a normalization of heart function (ejection fraction = 78%) following two months of dietary intervention (using solely the MCTs formula), with only minor left ventricular hypertrophy and no pericardial effusion (81). However, supplementation with KBs could offer a promising alternative dietary treatment strategy, since LCTs restriction and MCTs supplementation do not resolve muscle symptoms. The KBs acetoacetate (AcAc) and beta- hydroxybutyrate (BHB) can be quickly used as oxidative substrate by tissues as cardiac and skeletal muscle, but not by the liver. Studies in dogs have shown that the myocardium almost completely shifts from fat oxidation to BHB oxidation upon infusion of BHB (86, 87).

In the LCHADD population described by Kwiatkowska et al. every child followed a diet with a limited long-chain fatty acid intake (10% of total energy), MCTs supplements (10%–20% of energy) and standard HF medication when needed (74). During follow-up, the echocardiographic abnormalities of four children with DCM at the time of the LCHADD diagnosis normalized (74). Nasogastric tube feeding was frequently used and L-carnitine supplements were administered to children who had a proven L-carnitine deficit (74, 88, 89).

Immonen et al. described a series of six LCHADD patients who survived more than six months and were treated with a strict dietary regimen. Of these, four patients with initial cardiomyopathy normalized within two to six months of starting (84). Only one patient with a normal cardiac function developed mild cardiomyopathy during 10-year follow-up. However, a poor dietetic compliance was reported, contrary to the other patients (84). The dietary regimen was characterized by a restricted long-chain fat intake (minimum 2.6%, maximum 10.7%), by a general maximum fasting of 2–4 h and by the use of night feeding through gastrostomy or nasogastric tube (84).

Moreover, triheptanoin, a highly purified 7-carbon chain triglyceride, has recently been approved by the US FDA for treating LC-FAODs. Its metabolism provides both acetyl CoA and propionyl CoA, unlike MCTs oils that supply only acetyl CoA. Triheptanoin is recommended at 25%–35% of daily calories, while traditional MCTs oils are recommended at 15%–25% (73, 90). Triheptanoin lowers hospitalizations and significant clinical events (such as hypoglycemia, cardiomyopathy, and rhabdomyolysis) in patients with LC-FAODs (91). It is important to underline the positive effect on cardiac function (91). Gillingham et al. conducted a double-blind, controlled study where patients assigned to receive triheptanoin had a 20% decrease in left ventricular wall mass, a 7.4% improvement in left ventricular ejection fraction, and a lower heart rate for the same workload, if compared with patients with C8 oil (both 20% of daily calorie intake) (91). The most common side effects of triheptanoin described is gastrointestinal symptoms (73, 90).

#### 3.2.6 Acyl-CoA dehydrogenase family member 9 deficiency

*Background:* acyl-CoA dehydrogenase family member 9 (ACAD9) is a mitochondrial flavoenzyme and one of the factors of complex I respiratory chain (92, 93). Its Acyl-CoA dehydrogenase (ACAD) activity is essential for beta-oxidation of acyl-CoA (94). Since it catalyzes the initial step of the FAO cycle (95), but its role in this biochemical pathway remains ambiguous. Organs like liver and central nervous system express high levels of ACAD, which is paramount for full FAO in these organs, while in other tissues, as heart and muscles, ACAD is less expressed and does not contribute significantly to FAO (94, 96, 97). In ACAD deficiency the serum acylcarnitine profile is normal (92). ACAD9 deficiency is one of the most common causes of mitochondrial oxidative phosphorylation disorders and is determined by pathogenic biallelic variants of the gene *ACAD9* (98),. Clinical manifestations are heterogeneous regarding organ involvement, age of onset, progression and severity of symptoms. In particular, the disease severity is related to the residual ACAD9 enzyme activity, whereas there is no correlation with residual ACAD9 protein levels (94, 99). The clinical presentation in ACAD9 deficiency includes lactic acidosis, neurological symptoms with development delay and a low IQ, hepatic disorders, cardiac diseases and myopathy which causes muscular weakness and easy fatigability. In addition, patients may present intrauterine growth retardation, renal disorders (tubulopathy), ophthalmic conditions (optic atrophy), and premature ovarian failure (100, 101).

*Cardiac involvement:* the cardiac involvement manifests with a wide variety of conditions. The most common is a severe, early-onset, HCM, frequently lethal during childhood. Otherwise, it consists in dilated or combined cardiomyopathy or only in isolated electrical abnormalities in near-asymptomatic children (102, 103).

*Dietary interventions:* according to some authors, treatment with high dosage riboflavin can improve symptoms without side effects in patients with ACAD deficiency and predominant myopathy and cardiomyopathy (99, 101, 104, 105). No specific dietary treatment is described. Early-onset patients (within the first year of life) have a significant better survival when treated with riboflavin (99). However, not all ACAD9-mutated patients with complex I deficiencies always respond to riboflavin treatment (100, 106).

Riboflavin is the precursor of flavin adenine dinucleotide (FAD) and flavin mononucleotide (FMN), cofactors for complex I and dehydrogenases involved in FAO, including ACAD. Riboflavin increases mitochondrial FAD concentration, supports FAD binding to ACAD9 improving its folding and stability and thus promotes complex I assembly and activity (100, 107). In addition, riboflavin reduces plasma lactate levels and improves clinical exercise tolerance, muscle strength and cardiac function. Interestingly, there is a great clinical response to riboflavin treatment on echocardiography, without resorting to heart transplantation in many cases (100–102). Differently from cardiac transplantation, high dosage riboflavin treatment improves not only heart function, but it also impacts the other organs affected in ACAD9 deficiency (e.g., muscular, neurologic, renal systems, etc.) (108).

#### 3.2.7 Ketone body defects

*Background:* 2-methylacetoacetyl-CoA thiolase (MAT) deficiency, also known as *β*-ketothiolase deficiency, is caused by mutations in the gene *ACAT1* and affects ketone body metabolism and branched-chain amino acid (BCAA) catabolism. MAT plays a role in two processes: the breakdown of isoleucine, producing propionyl-CoA and acetyl-CoA, and the utilization of KBs, converting acetoacetyl-CoA into acetyl-CoA. Patients typically experience ketoacidotic crises between 6 and 36 months but remain asymptomatic between episodes. The clinical presentation can resemble ketotic hypoglycemia (1). 3-hydroxy-3-methylglutaryl-CoA lyase (HMGCL) deficiency is both an OA affecting leucine metabolism and a defect of KBs synthesis (acetoacetate and 3-hydroxy-n-butyrate). Patients with HMGCL deficiency usually show symptoms in the neonatal period, such as vomiting, seizures, and acute metabolic issues (1).

*Cardiac involvement:* As of now, 244 cases of MAT deficiency have been reported, with one known case of cardiac complications: an 8-year-old girl who died from HF due to severe DCM (109). A recent meta-analysis of 211 HMGCL deficiency cases revealed that cardiac issues were reported in a few cases, including DCM and cardiac arrest in one patient (110, 111). The first case of fatal arrhythmia linked to cardiomyopathy in HMGCL deficiency was reported in a 7-month-old boy by Gibson et al. (112). Leung et al. described a 23-year-old male with HMGCL deficiency who developed acute HF due to DCM (113). Köksal et al. reported a case of LVNC in an 8-month-old with HMGCL deficiency who presented with vomiting, respiratory distress, and seizures (2, 114).

*Dietary interventions:* dietary guidelines are not available. In MAT deficiency dietetic treatment consists in limited fasting time, low fat and low protein (2 g/kg/day) with low leucine intake and L-carnitine supplementation (115, 116). Similarly in HMGCL deficiency it is recommended to avoid fasting, limit protein intake (1.5–2 g/kg/day) and avoid a high-fat diet (117). No studies have been found on the effect of diet on cardiac function.

### 3.3 Cell processing and trafficking defects

#### 3.3.1 Congenital disorders of glycosylation

*Background and cardiac involvement:* Glycosylation is the process through which glycans are attached to proteins and lipids, crucial for their function (i.e., protein folding, solubility). CDG are multi system disorders, including neurological symptoms, cardiac, renal, liver, and gastrointestinal impairment, endocrine abnormalities, growth retardation and visual and hearing loss (118). In the heart, glyconjugates play key roles in signal transduction, depolarization, and cell adhesion. Cardiac impairment is present in several CDG even though it is less frequent than other organ involvement (2). These disorders are classified into four main biochemical categories: three related to protein glycosylation (N-linked glycosylation, O-linked glycosylation, and combined N- and O-glycosylation) and one involving lipid glycosylation (33, 119). The most common biochemical screening tools for CDG diagnosis, besides genetic sequencing, are plasma transferrin isoelectric focusing and mass spectrometry-based glycomics.

- **Phosphomannomutase 2 (PMM2) deficiency**: it was the first CDG to be described (120). It is an AR disorder caused by mutations affecting the gene *PMM2*, which encodes phosphomannomutase 2, a central enzyme in the mannose metabolism. PMM2-CDG clinical spectrum is primarily characterized by neurological manifestations (such as developmental delay, intellectual disability and seizures), ocular defects, endocrine abnormalities and failure to thrive. Cardiomyopathy, especially hypertrophic, is frequent and significantly impacts mortality of these patients (118). While some patients with isolated cardiomyopathy rapidly deteriorate, others may remain stable on supportive medication (angiotensin-converting enzyme inhibitors, *β*-blockers) (33). Cardiac failure, tamponade and pericardial effusions are adverse events reported in these patients from the prenatal/neonatal period to late childhood (10).
- **Phosphoglucomutase 1 (PGM1) deficiency:** it is an AR disorder caused by mutations affecting the gene *PGM1*, encoding phosphoglucomutase 1, a central enzyme in the glucose and glycogen metabolism and involved in N-glycosylation. PGM1-CDG affects multiple organs, and molecularly causes a decrease of galactosylation and impaired glycan synthesis (121). PGM1 deficiency causes a decrease in galactosylation and a general reduction in glycan synthesis (122). The most frequent clinical manifestations include hypoglycemia, liver dysfunction, growth retardation, and hypogonadotropic hypogonadism with delayed puberty. It has been estimated that approximately 50% of these patients develop DCM, with some cases progressing to cardiac arrest or requiring heart transplantation (121). Most patients also experience exercise intolerance, muscle weakness, and rhabdomyolysis (33); intellectual development is typically normal (122).
- **Fukutin (FKTN) deficiency:** this disorder can be classified as both an AR CDG and a congenital muscular dystrophy, caused by mutations on the gene *FKTN*, which encodes fukutin, a key enzyme in the glycosylation of a large protein named alpha-dystroglycan, which is essential for heart, muscle and brain functions. FKTN-CDG is clinically associated with mental disability and ocular involvement (123). It was demonstrated that FKTN can transfer a ribitol phosphate group confirming its role in the synthesis of O- mannosylglycan (124). Certain FKTN-CDG variants, specifically p.Q358P and p.R179T, are linked to DCM, typically with mild muscle weakness appearing in the second decade of life**.** Among six reported patients from four families, one died at age 12 due to DCM, and another received a heart transplant at age 18 (123). DCM with lymphocytic infiltration and fibrosis and myocardial fibrosis predominant in the left ventricular wall were reported by postmortem or explanted heart analysis (123).
- **Fukutin-related protein (FKRP) deficiency:** this AR disorder can also be classified as a CDG and as a of congenital muscular dystrophy, caused by mutations affecting the gene *FKTN*. This gene encodes the so called fukutin-related protein, a protein that, along with fukutin, contributes to the glycation of alpha-dystroglycan (124). The main clinical symptoms are limb-girdle muscular dystrophy, calf hypertrophy, elevated serum CPK as well as cardiac disease in many patients (123). Several patients were reported with cardiac involvement: conduction abnormalities (such as paroxysmal atrial fibrillation, incomplete right bundle branch block, polymorphic ventricular extrasystoles), left ventricular dysfunction with congestive HF, myocyte hypertrophy, increased ventricular wall thickness and interstitial fibrosis with extensive fatty replacement (123).

*Dietary interventions:* a recent review (122) focused on the currently available nutritional therapies for CDG, highlighting that only a few CDG are currently treatable. No studies have specifically evaluated its effect on cardiac function.

***Mannose supplementation:*** mannose serum concentration increases and consequently promotes its uptake into the cytosol, where it is converted to mannose-6-phosphate via an MPI (mannose phosphate isomerase) independent pathway (125). A dosing regimen of approximately 0.1–0.2 g/kg body weight every 4 h, administered 3–5 times daily, has been suggested (126). However, this should be considered only as a general guideline, with the exact dose tailored individually based on the patient's biochemical and clinical response over several weeks. Although mannose supplementation showed improvement in glycosylation *in vitro* studies, clinical evidence has not confirmed any therapeutic benefit. Therefore, mannose therapy is currently not considered effective for PMM2-CDG (122). Conversely, in MPI-CDG, which typically shows no cardiac involvement, coagulation abnormalities and hyperinsulinism generally improve over time (122)**.**

***D-galactose supplementation:*** based on positive *in vitro* responses of PGM1-CDG patient fibroblasts to D-galactose supplementation, clinical trials have been initiated. Preliminary results show improvements in liver enzymes, coagulation factors and some endocrine markers. The frequency of hypoglycemic and rhabdomyolysis episodes decreased with treatment, although muscle weakness and elevated CPK levels remained unchanged (122). Yet, not clear effects on DCM and cardiac functions have been reported so far.

***Ribose supplementation:*** ribose can increase cellular levels of CDP-ribitol, with potential therapeutic benefits (127). The use of orally administered ribose over a six-month period in a patient with muscular dystrophy caused by a mutation in the *FKRP* gene led to a significant reduction in CPK levels, reduced fatigue and pain, and improved muscle strength (127). To date, no studies have described supplementation of ribose in patients with CDG and cardiac involvement. However, positive effects of ribose supplementation in patients with HF patients with normal systolic function but impaired diastolic function, have been reported in literature (128). By restoring ATP levels, ribose improves diastolic function, which is highly dependent on cellular energy (128, 129).

## 4 Discussion

**Figure 3:**
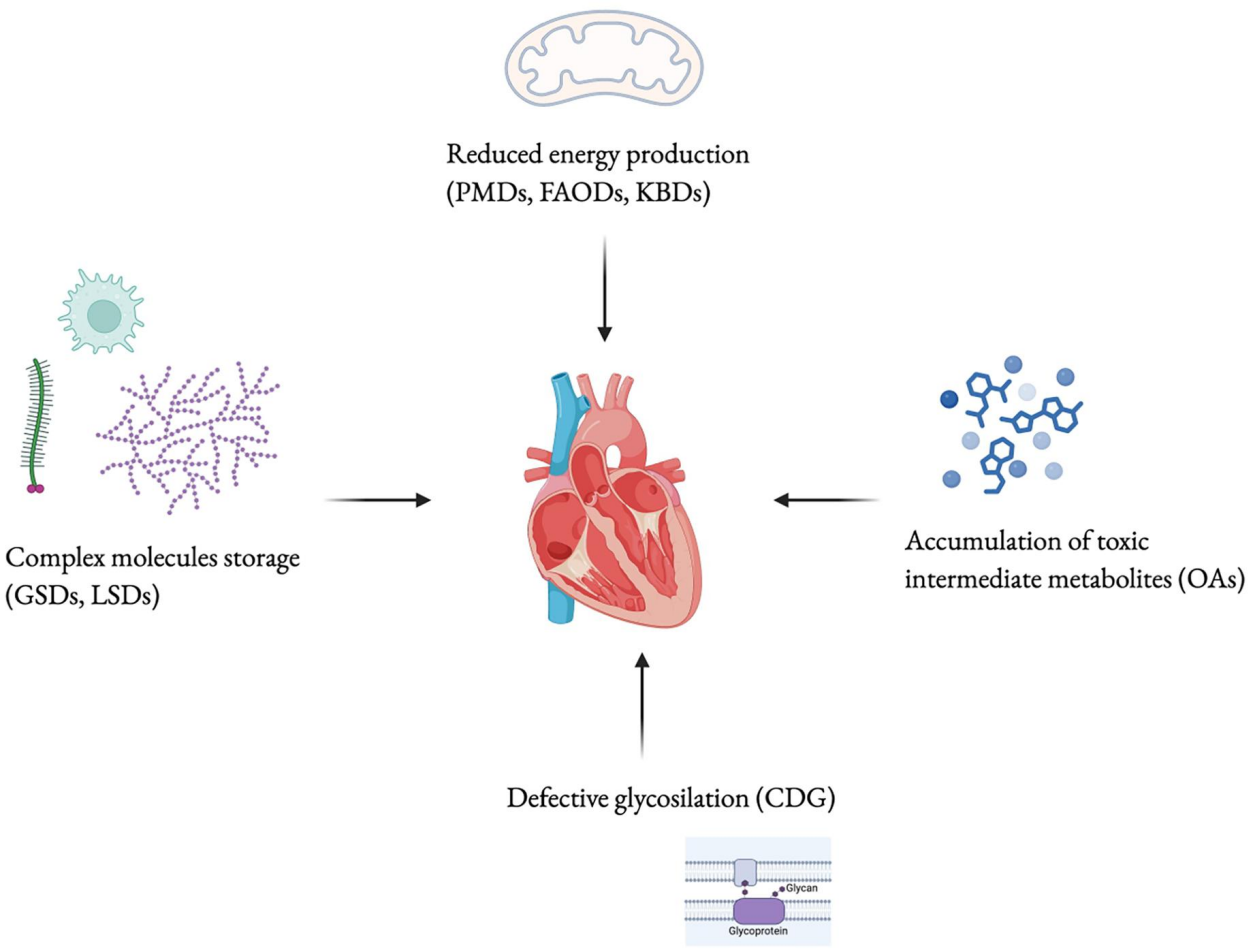
Cardiac damage pathophysiological mechanisms. PMDs, primary mitochondrial diseases; FAODs, fatty-acid oxidation disorders; KBDs, ketone body defects; GSDs, glycogen storage diseases; LSDs, lysosomial storage disorders; CDG, congenital disorders of glycosilation; OAs, organic acidurias. Created using Biorender.

**Figure 4:**
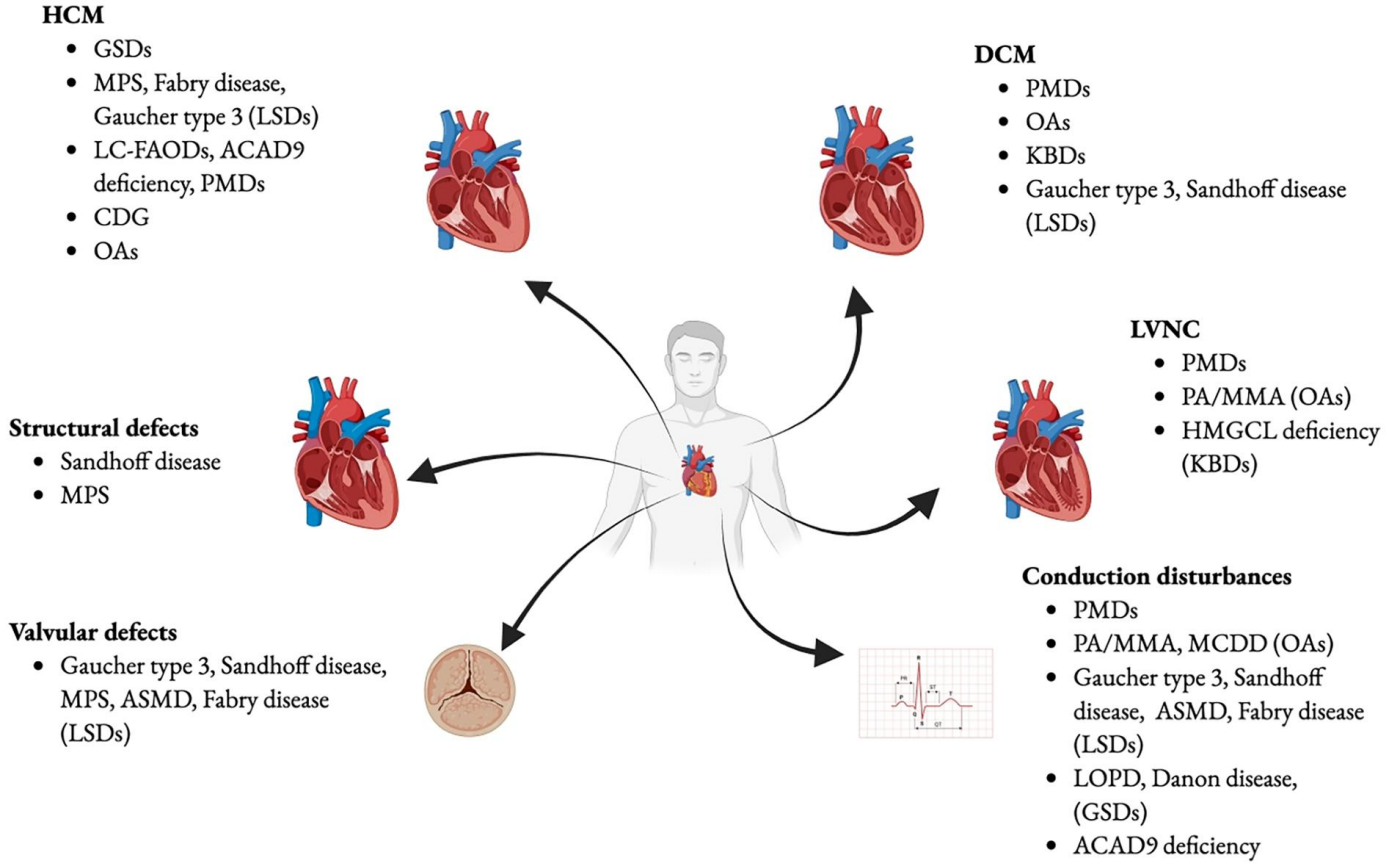
Cardiovascular diseases in IEMs. GSDs, glicogen storage diseases; MPS, mucopolisaccaridosis; LSDs, lysosomal storage disorders; LC-FAODs, Long-chain fatty-acid oxidation disorders; ACAD9, Acyl-CoA dehydrogenase family member 9; PMDs, primary mitochondria! diseases; CDG, Congenital disorders of glycosylation; OAs, organic acidurias; ASMD, Acid Sphingomyelinase deficiency; PA, propionic acidemia; MMA, methylmalonic acidemia; HMGCL, 3-hydroxy-3- methylglutaryl-CoA lyase; KBDs, ketone body defects; MCDD, Malonyl coenzyme A decarboxylase deficiency; LOPD, late onset Pompe disease. Created using Biorender.

Approximately 10% to 30% of the known causes of cardiomyopathy in childhood are attributable to IEMs (10, 130, 131). In IEMs, cardiac manifestations can be indicative symptoms discovered during regular multisystem screening. While in disorders like MPS, heart manifestations may dominate the clinical presentation, in others, such as PD, they represent the sole clinical manifestation. Four fundamental mechanisms underlie the pathophysiology of cardiac involvement. First, cardiac symptoms can be linked to a reduction in energy production resulting from genetic mutations in proteins involved in energy homeostasis, molecular transport, or cellular organelles. Second, the intracellular accumulation of intermediates or storage substrates within cardiac myocytes can lead to structural and functional damage of the cardiac tissue. Third, the accumulation of intermediate metabolites may exert toxic effects on cardiac and surrounding tissues, for example, by triggering apoptosis in cardiac myocytes. Fourth, altered cellular functions such as signal transduction, depolarization, and cell adhesion, caused by the absence or alteration of glyconjugates, can compromise tissue integrity and cardiac function. It is important to note that pathogenetic mechanisms, summarized in Figure 3, may often overlap, particularly in later stages of the illness progression (33). In this review, we offered a comprehensive description of the cardiovascular diseases primarily associated with various types of IEMs, to guide cardiologists in the differential diagnosis (Figure 4). Moreover, the diagnosis of an underlying metabolic disorder should rely on the recognition of associated signs and symptoms characteristic of each specific disease.

**Figure 5:**
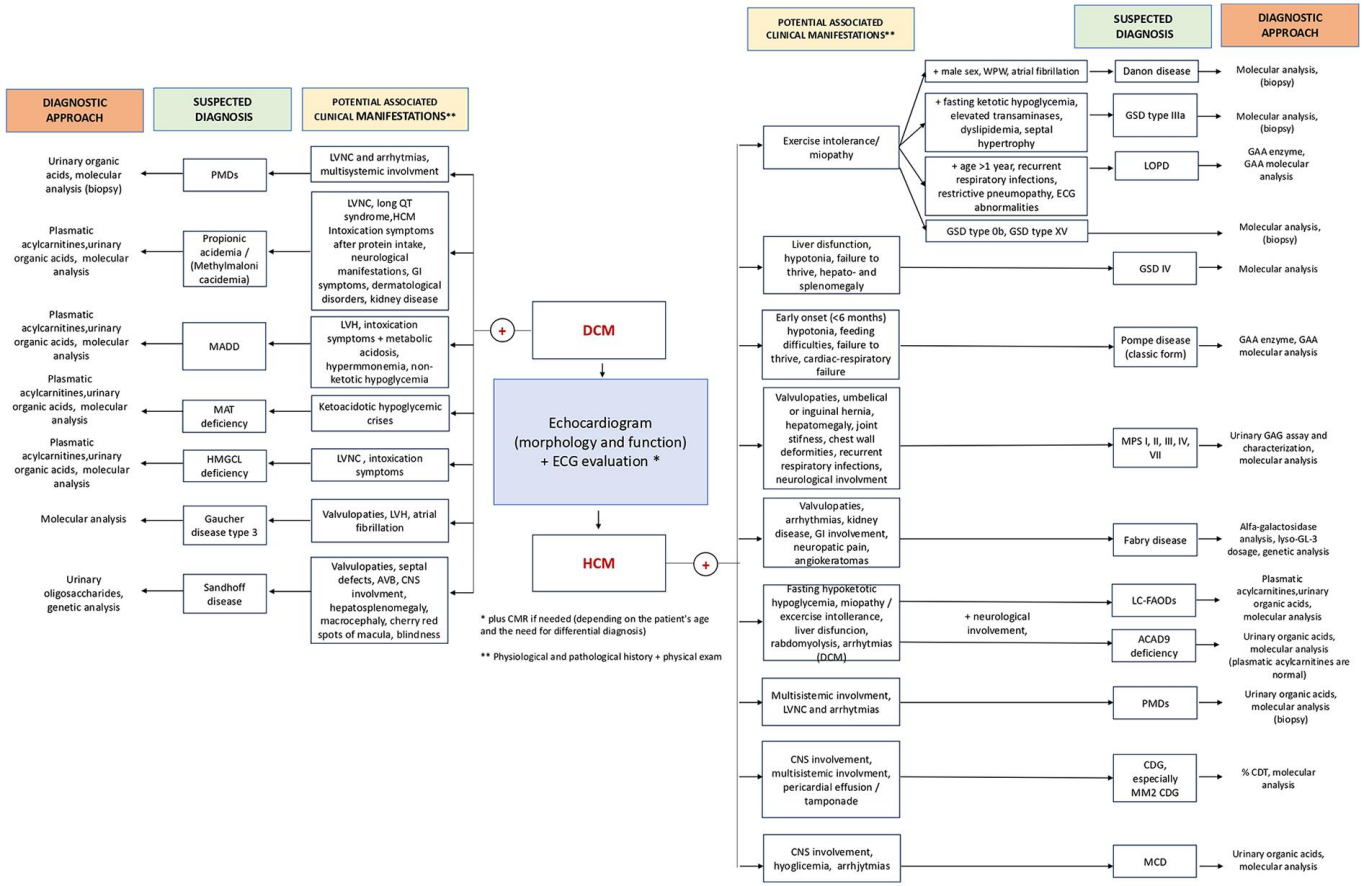
Approach to differential diagnosis. ACAD9, ACAD9D, Acyl-CoA dehydrogenase 9; AVB, atrio-ventricular block; CDG, congenital disorders of glycosilation; %CDT, % of carbohydrate-deficient transferrin; CNS, central nervous system; DCM, dilated cardiomyopathy; ECG, electrocardiogram; GAA, add alp ha-glucosidase; GAG, glycosaminoglycan; GI, gastrointestinal; GSD, glycogen storage disease; HCM, hypertrophic cardiomyopathy; HMGCL, 3-hydroxy-3-methylglutaryl-CoA lyase; LC-FAODs, Long-chain fatty-acid oxidation disorders; LOPD, Late onset pompe disease; LVH, left ventricular hypertrophy; LVNC, left ventricular non-compaction; MADD, Multiple acyl-CoA dehydrogenase deficiency; MAT, 2-methylacetoacetyl-CoA thiolase; MCD, malonyl coenzyme A (CoA) decarboxylase; MM2, phosphomannomutase 2; MPS, mucopolisaccaridosis; PMDs, primary mithocondrial diseases; WPW, Wolff-Parkinson-White.

IEMs have a wide phenotypic spectrum and may be characterized by a late onset or mild organ involvement, remaining misdiagnosed. Following the diagnosis of heart complications, the cardiologist should first conduct a detailed investigation of the patient's and family's medical history, including an assessment of consanguinity and/or the presence of rare inherited disorders. The patient's history should include age of onset of each clinically relevant symptom, the presence of associated pathological conditions and/or symptoms (hypoglycemia, myalgia, neurological issues or liver problems) and the result of neonatal screening. Physical examination is essential to assess for signs such as hepatosplenomegaly, inguinal or umbilical hernias, or other organ-related abnormalities that may suggest a specific diagnosis. Figure 5 can be useful to guide clinicians in the differential diagnosis of the two main types of cardiomyopathies encountered (HCM and DCM) based on potential associated signs and symptoms, including cardiac ones. It also serves as a guide for selecting appropriate second- and third-level diagnostic tests to confirm the suspected metabolic disorder. On the other hand, all clinicians involved in the care of patients with IEMs should be aware of the possible presence of a cardiac pathology, to guarantee an early diagnosis and intervention. The cardiological approach to paediatric cardiomyopathies in metabolic disease will combine, in addition to history and clinical evaluation, electrocardiography, two-dimensional and Doppler echocardiography and, if possible, ambulatory ECG monitoring, exercise testing and contrast-enhanced cardiac magnetic resonance (CMR). The European guidelines recommended CMR at initial evaluation (IB) and during follow-up (IIaC). Some ECG features, echocardiography pattern and CMR can help in suggesting specific aetiologies, but a detailed analysis of these patterns is beyond the scope of this paper (132).

Heart disease as the first presenting manifestation in some IEMs has been described. Sabate Rotes et al. described 12 patients with cardiomyopathy and IEMs, of which 50% had cardiac symptoms at the onset of the disease, which allowed an earlier diagnosis of the underlying metabolic disorder (133). Ten patients showed ventricular hypertrophy, associated with mitochondrial and lysosomal diseases, while two patients showed ventricular dilation, related to alterations in fatty acid metabolism (133). ACAD9 variants are the most frequent cause of cardiac hypertrophy and isolated complex I deficiency in childhood. In the presence of any, even mild, paediatric cardiac manifestation, and in case of early onset cardiac hypertrophy combined with elevated serum lactate levels, ACAD9 mutation should be investigated (134, 103, 102). Cardiac involvement, though rare, can be an early manifestation in Sandhoff disease during infancy (66). In VLCADD heart rhythm disorder could be the presenting symptom, thanks to which the diagnosis of the underlying inborn error of metabolism was made (10). Fortunately, in several states, newborn screening permits diagnosis of certain IEMs, such as LC-FAODs, before cardiac symptoms onset. On the other hand, a regular cardiological monitoring is crucial also for known LC-FAODs patients, to prevent further cardiac complications and death, in addition to avoiding triggering conditions. Kwiatkowska et al. described 2 LCHADD patients with DCM and HF, revealed by autopsy (74), underlining that, although cardiomyopathy is not the primary feature of LCHADD, echocardiography should be performed regularly to enable early detection. Moreover, CMR in patients with normal echocardiograms could provide further insights into this issue. Further research and multicentre clinical trials are needed to gain a deeper understanding (74). Additionally, screening for CDG should be performed on pediatric patients with undetected cardiomyopathy and echocardiography should be used to periodically check for cardiac problems in patients with CDG type Ia (10).

Secondarily, we reviewed the different dietary approaches used in IEMs, emphasizing their application or adaptation according to the underlying heart disease. In the absence of specific guidelines, we described case reports from the most recent literature to provide an idea of targeted dietary treatments on cardiac issues, in the contest of the IEMs (Table 1).

**Table 1:** Dietary treatments Inborn Errors of Metabolism.

Inborn Errors of Metabolism	Specific diesease	General dietary treatments	Dietary interventions with reported beneficial effects on cardiac function
Organic acidemias (OAs)	Propionic aciduria (PA)	Protein intake restriction; isoleucine/valine supplementation (12) L-carnitine supplementation (7) Micronutrients (zinc, copper, selenium, and iron) supplementation (17) Vitamin B12 in responsive MMA (12)	L-3-hydroxybutyrate supplementation (15) CoQ10 supplementation (1.5–25 mg/Kg/day) (15)
	Metilmalonic		
	Aciduria (MMA)		
	Malonyl coenzyme A decarboxylase deficiency (MCD)	Low-fat/high-carbohydrate diet (20)	High MCTs/low LCTs (20, 22) Carnitine supplementation (22)
	Multiple acyl-CoA dehydrogenase deficiency (MADD)	Low protein/fat diet (19) Limitation of prolonged fasting (19) Riboflavin in responsive forms (19) CoQ10 and carnitine supplementation in secondary deficiency (19)	L-3-hydroxybutyrate supplementation (18)
Glycogen storage diseases (GSDs)	Pompe disease (GSD type IIa)	High-protein, low-carbohydrate diet (44) L-alanine supplementation (46, 47)	L-alanine supplementation (46, 47)
	Cori-Forbes disease (GSD type IIIa)	Frequent meals, raw cornstarch use (35%–55% of total energy by carbohydrates, 20%–30% by proteins and 20%–35% by lipids) (34)	High-protein, low-carbohydrates diet (31, 34, 35) Low-calories (900 kcal/day), high-protein diet (37%–43% of total energy) (36) High-fat (50%), high-protein (20%), low-carbohydrates (30%) diet (31) High-fat (60%) high-protein (25%), low-carbohydrate (15%) diet (39) High-fat (70.5–75.7%), high-protein (18–28%) diet (42) Classic ketogenic diet (38) Ketogenic (2:1), high protein (15%) diet and synthetic ketone bodies (D,L-3-OH butyrate) supplementation (40) Modified Atkins diet (MAD) (30) MCTs supplementation/replacement (41)
Lysosomal storage diseases (LSDs)	Gaucher disease	Vitamin D and calcium supplementation (52) Limiting consumption of disaccharides (5)	Not reported
	Acid sphingomyelinase deficiency (ASMD)	No specific indications for low-fat diet	Not reported
	Fabry disease	Low-FODMAP diet (5, 26) (in case of GI symptoms) Low-protein diet supplemented with keto analogues (in case of CKD) (26)	Not reported
	GM2 gangliosidosis (Sandhoff disease)	Not reported	Ketogenic diet (64)
	Mucopolysaccharidoses (MPS)	Supplementation with vitamins B1, B2, B3, vitamin C and iron (5, 26) Fat-rich diet (59, 60) Supplementation with CoQ10 and PLP (62, 63)	Not reported
Fatty acid beta-oxidation disordes (FAODs)	LC-FAODs	VLACADD—LCHAD • Avoid prolonged fasting, LCTs intake restriction, MCTs supplementation, moderate increase of carbohydrates (71) • Triheptaoin recommended at 25–35% of daily calories (71, 81, 82) PCD • L-carnitine supplementation (74) ACAD9 deficiency • Riboflavin (94, 96, 99, 100)	Triheptaoin recommended at 25%–35% of daily calories (71, 81, 82)
			VLCADD: • MCTs + riboflavin + high IV dextrose (76)
			LCHADD: • High MCTs/low LCTs + carnitine and short fasting periods and continuous nocturnal feeding (72) • Riboflavin (94, 96, 99, 100)
Primary Mitochondrial Disease (PMDs)		Ketogenic diet (5, 68, 69)	Ketogenic diet (5, 68, 69)

AcAc, acetoacetate; ACAD, Acyl-CoA dehydrogenase; ACADD, Acyl-CoA dehydrogenase deficency; ASMD, Acid sphingomyelinase deficiency; BHB, Beta-hydroxybutyrate; CKD, Chronic kidney disease; FAODs, Fatty acid B-oxidation disordes; GSDs, Glycogen storage diseases; IV, intravenous; LCTs, long-chain triglycerides; LC-FAODs, Long-chain Fatty acid B-oxidation disordes; FAODs, Fatty acid B-oxidation disordes; GI, gastrointestinal; LSDs, Lysosomal storage diseases; MAD, Modified Atkins diet; MADD, Multiple acyl-CoA dehydrogenase deficiency; MCD, Malonyl coenzyme A decarboxylase deficiency; MCTs, medium-chain triglycerides; MMA, Metilmalonic Aciduria; MPS, Mucopolysaccharidoses; OA, Organic acidemias; PA, Propionic aciduria; PCD, Primary Carnitine deficiency; PMDs, Primary Mitochondrial Disease; PLP, pyridoxal phosphate; VLCADD, very-long-chain acyl-CoA dehydrogenase deficiency.

Generally, cardiac cells prefer and respond better to KBs over glucose for energy, even when both glucose and KBs are available (74). Based on this evidence, the common goal of the several dietary treatments described above in IEMs is to provide an alternative energy source, achieving a therapeutic ketosis, which allows bypassing defective metabolic pathways. While dietary strategies may differ, such as high fat diet, use of MCTs, synthetic KBs, they share the same objective. On the contrary, EMs in which ketosis is contraindicated are ketogenesis and ketolysis defects, where a low-fat diet is recommended.

KD has been implemented to be an available treatment in patients with IEMs and heart complications. The specific ratio of fat, protein, and carbohydrates can vary, but typically less than a third of calories come from protein and carbohydrates, while the majority comes from fat. MAD is a less restrictive version that limits carbohydrates but allows more flexibility with fats and proteins, making it easier for patients, especially children, to follow (5).

GSD III represents the pathology where these kinds of diets have been most widely used for the treatment of the associated cardiomyopathy. Several case reports have been described in the results with positive outcomes in reversing cardiomyopathy and maintaining normoglycemia. Moreover, chronic ketosis can lower insulin levels and decrease glycogen disposition (41).

The study by Rossi et al. asserts that a high fat diet could be an alternative dietary treatment in paediatric GSD III patients with cardiomyopathy (45). However, the improvement in muscle strength reported in GSD III should be objectified, and the role of dietary lipids remains a topic of debate for potential side effects including growth limitation, liver inflammation, and the development of hepatocellular carcinoma (45). Few data are available about the effect of high fat diet on transaminases: 44% of paediatric GSD III patients showed ALT increase, while decreased in adult patients (43). Moreover, it is important to closely monitor the long-term effects of a high fat diet on bone condition because of the increased risk of osteoporosis (45).

Since there is currently no standard and curative treatment for mitochondrial diseases, KD is an alternative diet also for PMDs, thanks to its capacity, demonstrated in animal and cell models, to reduce oxidative stress, boost antioxidants, and scavenge free radicals (135).

The reason for this benefit has been demonstrated by the good response of the heart and brain tissue to the fuel provided by KBs. KD is believed to benefit mitochondrial function by enhancing mitochondrial biogenesis, reducing oxidative stress, and providing energy through ketones (5). Hyperketonaemia is also linked to positive cardiovascular effects, which may be brought about by better cardiac energetics and lower oxygen consumption, both by preventing and treating cardiomyopathy (136). Nevertheless, the positive cardiovascular effects of KD may be offset by hyperlipidaemia, which is characterized by elevated levels of LDL cholesterol and triglycerides (136), with possible increased risk of atherosclerosis. Other side effects may be bradycardia, constipation and headaches (5).

There is an emerging use of MCTs as a high-speed energy source, due to their tendency to be more oxidized than stored, since mitochondrial transport is not carnitine dependent (45). Use of MCTs can be another dietetic strategy, as these can be converted into KBs by the liver but also they can be used as an alternative energy substrate. MCTs can be used within a high fat diet, such as in GSD III or as a replacement for LCTs in LC-FAODs.

Concerns regarding MCTs use in GSDs patients are the uncertain impact on the elongation of fatty acids or the gluconeogenesis pathway (137). Moreover, there have been reports of elevated triglyceride levels in GSD III patients following the administration of MCTs (138). Hypertriglyceridemia and hypercholesterolaemia correlate negatively with age In GSD IIIa and may reflect increased severity of hypoglycaemia in this younger population (139).

MCTs are effective in treating LC-FAODs because they bypass the metabolic defect, can diffuse across the mitochondrial membrane without transporters, and undergo fewer rounds of beta-oxidation, feeding directly into the tricarboxylic acid cycle for energy production. They play a role in ameliorating cardiomyopathy, in a variable percentage and associated to limiting LCFAs. Adequate adherence to diet therapies and preventive treatment in acute conditions are essential to avoid clinical decompensation and worsening of cardiac function in patients with cardiomyopathy. Echocardiographic improvement has also been demonstrated in patients followed up with a strict diet (81, 84).

Moreover, cases of improved cardiac function in patients with MCD deficiency after a long-chain triglycerides (LCTs)-restricted/MCTs-supplemented diet have been described (24, 26).

Finally, the direct supplementation of synthetic KBs has been reported. Some experiences of synthetic KB use are reported in PA or MADD, with cardiac improvement (16, 22). In these studies, the authors suggested that this improvement was due to enhanced energy availability in the heart through KBs, without relying on FAO.

SGLT2 inhibitors are antidiabetic drugs that improve glycemic control and promote weight loss. They may increase the risk of diabetic ketoacidosis through several biologically plausible mechanisms, including reduced insulin dosing, enhanced glucagon secretion, and decreased renal clearance of KBs. Generally, SGLT2 inhibitors reduce cardiovascular death and HF hospitalization in high-risk patients both with and without diabetes, improving outcomes in HF with reduced and preserved ejection fraction through hemodynamic, anti-inflammatory, and metabolic benefits beyond glucose lowering (140). The ability of SGLT2 inhibitors to induce moderate ketosis may represent a potential therapeutic strategy in IEMs where promoting ketosis could help improve cardiomyopathy (141). Moreover, empagliflozin has been successfully repurposed for treating neutropenia and neutrophil dysfunction in patients with glycogen storage disease type 1b (GSD Ib) (142).

Not only ketosis has been described as effective in cardiac function improvement. An improvement of cardiomyopathy has been described in GSD III patients after a high protein diet. When glycolysis is impaired, like in GSD III, a high protein diet can be an alternative source of glucose during fasting, to increase gluconeogenesis, since gluconeogenesis is intact in GSD III. In addition, muscle function can be improved thanks to an increased muscle protein synthesis and glycogen accumulation decreases. Preventing carbohydrate overtreatment and introducing a high-protein diet can reverse or avoid cardiomyopathy. Supplementation with a single amino acid, such as L-alanine, has also been reported to be effective in improving muscular and cardiac function in patients with PD (48, 49).

Research on nutritional management in LSDs, especially in case of cardiomyopathy, remains scarce. Only in Sandhoff disease KD and its effect on heart is described.

While there are no specific dietary recommendations for GD patients, it is crucial to monitor their nutritional and metabolic status to develop an appropriate dietary plan tailored to their individual needs. However, assessing the effectiveness of nutritional therapy is challenging due to the potential influence of lifestyle factors and ERT or SRT on metabolism, which are not yet fully understood (29).

Only a few CDG are currently treatable and no studies about impact of dietary interventions on cardiac function have specifically evaluated. It is interesting to note the potential positive impact of ribose supplementation on cardiac function, as suggested by several studies. Ribose has been reported to improve cardiac dysfunction after ischemia (143). Ribose has demonstrated an ability to replenish low myocardial energy levels, improving diastolic dysfunction and quality of life of patients with congestive HF (144, 145). Moreover, ribose supplementation led to significant improvements in ventilation efficiency in patients with HF, demonstrating its potential to improve exercise capacity (143, 144). This finding may support the potential future use of ribose in CDG patients experiencing diastolic dysfunction.

Finally, in patients with IEMs, liver transplant is one of the options being considered to improve the metabolic control and compensation of patients. As some of these conditions carry a risk of cardiac involvement, their impact on the heart could be significant. For instance, in PA, cardiomyopathy is one of the major complications and liver transplant, which is indicated in pediatric patients with recurrent metabolic decompensation, has been described to reduce mortality, reduce decompensation episodes, and even reverse cardiomyopathy (9), without the necessity of a protein-restricted diet anymore (146).

However, not all manifestations of prolonged toxin accumulation are reversible. Two case reports (147, 148) showed the recurrence of cardiomyopathy years after liver transplantation, probably as a consequence of long exposure to toxic metabolites in the past. The literature contains limited long-term data on cardiac follow-up after liver transplantation (149). In selected cases of severe heart disease, a combined heart and liver transplantation can be considered (150), even if a strict multidisciplinary approach is needed to prevent surgical metabolic decompensation (151).

## 5 Conclusion

Paediatricians and cardiologists should always raise suspicion and investigate the possibility of an underlying inborn error of metabolism in patients with heart diseases, especially in conditions not included in newborn screening, such as PMDs, GSDs and some LSDs. Recent advancements in diagnosing and managing IEMs, particularly with the introduction of newborn screening and next-generation sequencing, have significantly improved our understanding of the cardiac manifestations in this heterogeneous group of congenital diseases. Newborn screening enables a pre-symptomatic diagnosis of IEMs and cardiomyopathy screening should be considered a standard of care during follow-up. As diagnoses increase, continued progress in treatment and management is crucial to reduce morbidity and mortality, including the development of new dietary approaches targeting cardiac involvement. In this clinically complex patient population, dietary treatment must be highly individualized and requires a multidisciplinary approach. Potential side effects of use of MCTs or KD should always be considered and carefully monitored. Further research is needed to explore the broader effects of these treatments in patients with IEMs and to identify those who are most likely to benefit from them.
