# Supplementary material for: An overview on cardiac involvement in Inborn Errors of Metabolism: from clinical clues to nutritional management strategies
Source: Front Cardiovasc Med. 2025 Dec 4;12:1648010. doi: 10.3389/fcvm.2025.1648010 (PMC12711851; doi:10.3389/fcvm.2025.1648010)
Supplement: Supplementary file 1 [file Table1.pdf]

Supplementary Table 1. PECO/PICO criteria for inclusion of studies.

TOPIC	Narrative question	P - Population	E/I – Exposure/Intervention	C - Comparison/Control	O - Outcomes
**Topic 1**<br>**Organic Acidemias**	1.1 In patients with Organic Acidemias, what are the main types of cardiac involvement?<br>1.2 Which dietary treatments improve cardiac function in patients with Organic Acidemias and cardiac involvement compared to standard management?	Infants, children, adolescents or adults with Organic Acidemias	E: Identification of the different types of cardiac involvement.<br><br>I: Targeted dietary treatment for Organic Acidemias with detected cardiac involvement.	No cardiac involvement or other types of involvement.<br><br>Absence of dietary treatment or non-targeted standard treatment.	Identified through:<br>- Type and severity of cardiac manifestations.<br>- Improvement of cardiac function or reduction in the progression of heart disease.
**Topic 2**<br>**Glycogen Storage Diseases**	2.1 In patients with Glycogen Storage Diseases, what are the main types of cardiac involvement?<br>2.2 Which dietary treatments improve cardiac function in patients with Glycogen Storage Diseases and cardiac involvement compared to standard management?	Infants, children, adolescents or adults with Glycogen Storage Diseases	E: Identification of the different types of cardiac involvement.<br><br>I: Targeted dietary treatment for Glycogen Storage Diseases with detected cardiac involvement.	No cardiac involvement or other types of involvement.<br><br>Absence of dietary treatment or non-targeted standard treatment.	Identified through:<br>- Type and severity of cardiac manifestations.<br>- Improvement of cardiac function or reduction in the progression of heart disease
**Topic 3**	3.1 In patients with Lysosomal Storage Diseases, what are the main types of cardiac involvement?	Infants, children, adolescents or adults with Lysosomal Storage Diseases	E: Identification of the different types of cardiac involvement.	No cardiac involvement or other types of involvement.	Identified through:<br>- Type and severity of cardiac manifestations.

**Lysosomal Storage Diseases**	3.2 Which dietary treatments improve cardiac function in patients with Lysosomal Storage Diseases and cardiac involvement compared to standard management?		I: Targeted dietary treatment for Lysosomal Storage Diseases with detected cardiac involvement.	Absence of dietary treatment or non-targeted standard treatment.	- Improvement of cardiac function or reduction in the progression of heart disease
**Topic 4**<br><br>**Primary Mitochondrial Diseases**	4.1 In patients with Primary Mitochondrial Diseases, what are the main types of cardiac involvement?<br><br>4.2 Which dietary treatments improve cardiac function in patients with Primary Mitochondrial Diseases and cardiac involvement compared to standard management?	Infants, children, adolescents or adults with Primary Mitochondrial Diseases	E: Identification of the different types of cardiac involvement.<br><br>I: Targeted dietary treatment for Primary Mitochondrial Diseases with detected cardiac involvement.	No cardiac involvement or other types of involvement.<br><br>Absence of dietary treatment or non-targeted standard treatment.	Identified through:<br><br>- Type and severity of cardiac manifestations.<br>- Improvement of cardiac function or reduction in the progression of heart disease
**Topic 5**<br><br>**Fatty Acid β-oxidation Disorders**	5.1 In patients with Fatty Acid β-oxidation Disorders, what are the main types of cardiac involvement?<br><br>5.2 Which dietary treatments improve cardiac function in patients with Fatty Acid β-oxidation Disorders and cardiac involvement compared	Infants, children, adolescents or adults with Fatty Acid β-oxidation Disorders	E: Identification of the different types of cardiac involvement.<br><br>I: Targeted dietary treatment for Fatty Acid β-oxidation Disorders with detected cardiac involvement.	No cardiac involvement or other types of involvement.<br><br>Absence of dietary treatment or non-targeted standard treatment.	Identified through:<br><br>- Type and severity of cardiac manifestations.<br>- Improvement of cardiac function or reduction in the progression of heart disease

	to standard management?				
**Topic 6**<br><br>**Ketone Body Defects**	6.1 In patients with Ketone Body Defects, what are the main types of cardiac involvement?<br><br>6.2 Which dietary treatments improve cardiac function in patients with Ketone Body Defects and cardiac involvement compared to standard management?	Infants, children, adolescents or adults with Ketone Body Defects	E: Identification of the different types of cardiac involvement.<br><br>I: Targeted dietary treatment for Ketone Body Defects with detected cardiac involvement.	No cardiac involvement or other types of involvement.<br><br>Absence of dietary treatment or non-targeted standard treatment.	Identified through:<br><br>- Type and severity of cardiac manifestations.<br>- Improvement of cardiac function or reduction in the progression of heart disease)
**Topic 7**<br><br>**Congenital Disorders of Glycosylation**	7.1 In patients with Congenital Disorders of Glycosylation, what are the main types of cardiac involvement?<br><br>7.2 Which dietary treatments improve cardiac function in patients with Congenital Disorders of Glycosylation and cardiac involvement compared to standard management?	Infants, children, adolescents or adults with Congenital Disorders of Glycosylation	E: Identification of the different types of cardiac involvement.<br><br>I: Targeted dietary treatment for Congenital Disorders of Glycosylation with detected cardiac involvement.	No cardiac involvement or other types of involvement.<br><br>Absence of dietary treatment or non-targeted standard treatment.	Identified through:<br><br>- Type and severity of cardiac manifestations.<br>- Improvement of cardiac function or reduction in the progression of heart disease)
